# The distinct metabolism between large and small HDL indicates unique origins of human apolipoprotein A4

**DOI:** 10.1172/jci.insight.162481

**Published:** 2023-04-24

**Authors:** Allison B. Andraski, Sasha A. Singh, Hideyuki Higashi, Lang Ho Lee, Masanori Aikawa, Frank M. Sacks

**Affiliations:** 1 Department of Nutrition, Harvard T.H. Chan School of Public Health, Boston, Massachusetts, USA.; 2 Center for Interdisciplinary Cardiovascular Sciences, Division of Cardiovascular Medicine, and; 3 Channing Division of Network Medicine, Department of Medicine, Brigham and Women’s Hospital, Harvard Medical School, Boston, Massachusetts, USA.

**Keywords:** Metabolism, Cardiovascular disease, Lipoproteins

## Abstract

Apolipoprotein A4’s (APOA4’s) functions on HDL in humans are not well understood. A unique feature of APOA4 is that it is an intestinal apolipoprotein secreted on HDL and chylomicrons. The goal of this study was to gain a better understanding of the origin and function of APOA4 on HDL by studying its metabolism across 6 HDL sizes. Twelve participants completed a metabolic tracer study. HDL was isolated by APOA1 immunopurification and separated by size. Tracer enrichments for APOA4 and APOA1 were determined by targeted mass spectrometry, and metabolic rates were derived by compartmental modeling. APOA4 metabolism on small HDL (alpha3, prebeta, and very small prebeta) was distinct from that of APOA4 on large HDL (alpha0, 1, 2). APOA4 on small HDL appeared in circulation by 30 minutes and was relatively rapidly catabolized. In contrast, APOA4 on large HDL appeared in circulation later (1–2 hours) and had a much slower catabolism. The unique metabolic profiles of APOA4 on small and large HDL likely indicate that each has a distinct origin and function in humans. This evidence supports the notion that APOA4 on small HDL originates directly from the small intestine while APOA4 on large HDL originates from chylomicron transfer.

## Graphical abstract

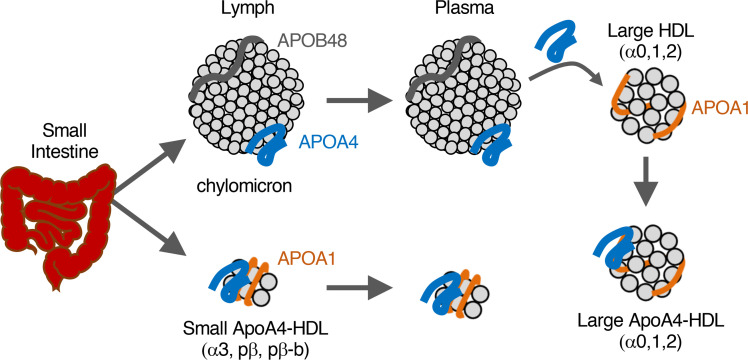

## Introduction

HDL-cholesterol in the blood is a strong inverse predictor of coronary heart disease (CHD) risk (1, 2). Pharmacological interventions that raise HDL-cholesterol levels, however, have failed to lower CHD (3–5). Measuring HDL-cholesterol alone does not reflect HDL’s diverse particle heterogeneity and function. Over 200 proteins have been associated with HDL (6). HDL particles also range in size from very small discoidal, cholesterol ester–poor prebeta to larger, spherical, cholesterol ester–rich alpha particles, including alpha3, 2, 1, and the largest, alpha0 (7–9). Protein-defined HDL subspecies each have their own distinct proteome and distribution across HDL size (7, 10–12) and are also differentially associated with risk of CHD, stroke, and diabetes (13, 14). Overall, HDL proteins as opposed to HDL-cholesterol levels may be better indicators of disease risk and targets for treatment.

The diverse array of protein-based and size-based HDL particles in circulation may in part be due to different organ-specific HDL synthesis. Both the liver and small intestine produce HDL (15). Studying small intestine–derived HDL has been challenging, as there is currently no way to distinguish it from liver-derived HDL in human plasma. However, apolipoprotein A4 (APOA4) is unique in that it is the only HDL apolipoprotein in humans that is primarily synthesized by the enterocytes of the small intestine, with only minor amounts (<5%) synthesized by liver hepatocytes (16–18). We thus hypothesize that APOA4 serves as a marker of small intestine–derived HDL and that studying its metabolism across multiple HDL sizes in humans will provide a better understanding of APOA4 and small intestine HDL origin and function in humans in vivo.

APOA4 in humans was first identified on the surface of chylomicrons (19, 20) and later on HDL as well as in the smaller density (>1.21 g/mL) lipoprotein-deficient plasma fraction (21, 22). In HDL proteomic studies, APOA4 is estimated to be among the top 10% of most abundant HDL proteins, with an abundance 10-fold less than that of apolipoprotein A1 (APOA1), the primary protein on HDL (11, 23). APOA4 is expressed by enterocytes but not by other small intestinal cell types (24), and its synthesis and secretion in humans are increased by a high-fat meal (25–27). Evidence in medium collected from the human intestinal cell line Caco-2 and in the perfusate collected using a rodent small intestine in situ perfusion model indicates that APOA4-containing HDL in the small alpha3 and prebeta HDL sizes are synthesized by the small intestine (15, 28, 29) and secreted into the circulation via the lymphatics (29). In humans, studies using radiolabeled APOA4 on chylomicrons injected into human plasma show that HDL with APOA4 can also be formed in circulation by transfer of APOA4 from chylomicrons to circulating HDL (30). Unlike APOA4 on chylomicrons, APOA4 appears to be a stable constituent of HDL (31). Thus, APOA4 may transfer to, but not from, HDL (30, 32).

Evidence from cell culture and animal studies has identified APOA4 as a key mediator of diverse biological functions: APOA4 activates chylomicron lipolysis (33–35), promotes reverse cholesterol transport from cells to HDL (36–38), protects LDL from oxidation (39), improves glucose homeostasis (40–42), suppresses appetite (43), decreases inflammation (44, 45), promotes clearance of beta-amyloid plaques (46), and inhibits platelet aggregation and thrombosis (47). Whether APOA4 orchestrates these same functions in humans, however, is less clear and remains to be elucidated.

Several studies have examined the metabolism of APOA4 in humans (7, 30, 32, 48–50). But only 2 studies have analyzed APOA4 metabolism specifically in total HDL (30, 32) and 1 in alpha3 and prebeta HDL, the HDL sizes in which APOA4 is most abundant (7). In the present study, we capitalized on technological advances of high-resolution targeted mass spectrometry (51) to determine the metabolism of APOA4 not only in alpha3 and prebeta but also in the larger HDL sizes (alpha0, 1, 2) in which APOA4 is approximately 100-fold less abundant than that of APOA4 on prebeta (7). Moreover, we were able to detect APOA4 tracer in a section of the nondenaturing gradient gel below that of prebeta, which we term herein as prebeta-b for “below prebeta,” and included this sixth HDL size fraction in our APOA4 kinetic analysis.

The overall aim of this study was to determine the metabolism of APOA4 across 6 HDL sizes. We also determined the metabolism of APOA1, the primary protein on HDL and a surrogate for HDL particles, across the 6 HDL sizes and compared it with that of APOA4. Determining APOA4 metabolism across multiple HDL sizes may provide a better understanding of APOA4 origin and function in humans in vivo and increase our understanding of the role of small intestine–derived HDL in regulating health and disease.

## Results

### Study design.

**Figure 1:**
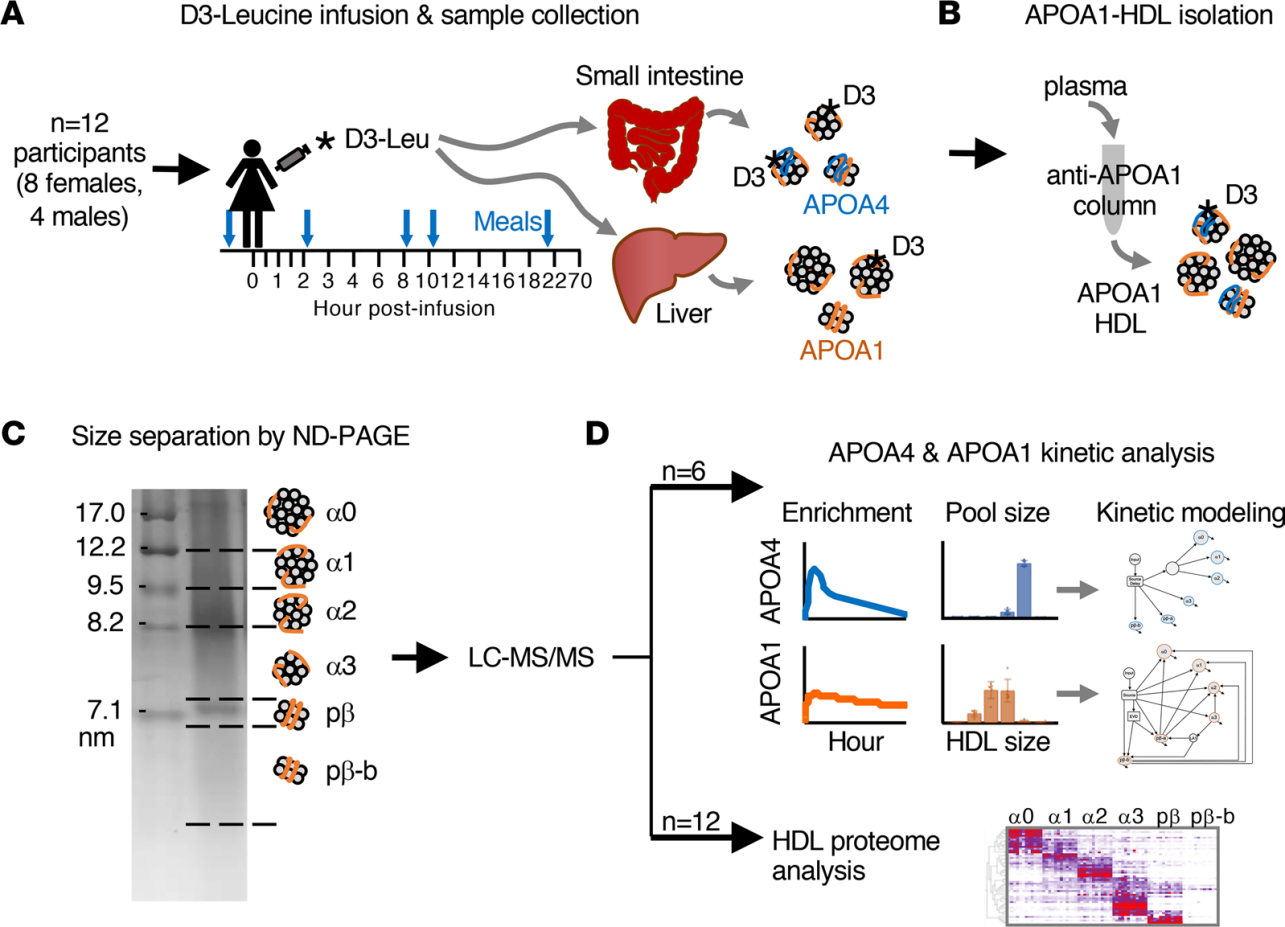
Study design overview. (**A**) A total of 12 participants were recruited and completed a metabolic tracer study. Participants consumed a controlled diet (40% fat, 45% carbohydrate, 15% protein) for 4 weeks prior to the tracer study and a low-leucine diet (38% fat, 56% carbohydrate, 6% protein) for 3 days during the tracer study. During the morning of the tracer infusion, participants consumed breakfast between 6 and 8 am before being admitted to the hospital at 9 am. At 10 am (time 0 hour) each participant was infused with a bolus of tri-deuterated leucine (D3-Leu). D3-Leu circulates throughout the body and incorporates into newly synthesized proteins in the small intestine (i.e., APOA1 and APOA4), liver (i.e., APOA1), and other organ systems. Blood samples were collected for 70 hours postinfusion (blood sample time points are shown as black lines on tracer infusion timeline). Participants consumed lunch, dinner, and a snack immediately following the 2-hour (12 pm), 8-hour (6 pm), and 10-hour (8 pm) blood draws, respectively. Breakfast the following morning was given at 7:15 am, before the 22-hour (8 am) blood draw (meals shown as blue arrows above tracer infusion timeline). (**B**) APOA1-HDL was isolated by anti-APOA1 immunoaffinity column chromatography for 1 to 14 time points per participant and (**C**) separated into 6 HDL sizes by nondenaturing polyacrylamide gel electrophoresis (ND-PAGE). Samples were then prepared for analysis by liquid chromatography-tandem mass spectrometry (LC-MS/MS). (**D**, top) APOA4 and APOA1 enrichments were quantified by targeted mass spectrometry, and absolute quantification of protein pool sizes were determined by stable isotope-labeled peptide standards for participants 1–6. These data were then used for kinetic modeling. (**D**, bottom) The HDL proteome was determined by global proteomics for the 6 APOA1-HDL sizes for all participants 1–12.

Twelve participants (Supplemental Table 1; supplemental material available online with this article; https://doi.org/10.1172/jci.insight.162481DS1) completed a metabolic tracer study (Figure 1A). HDL was isolated by anti-APOA1 immunopurification (Figure 1B), separated into 6 HDL sizes by nondenaturing polyacrylamide gel electrophoresis, and prepared for mass spectrometry analysis (Figure 1C). For participants 1 to 6, APOA1 and APOA4 tracer enrichment was determined by targeted mass spectrometry, pool sizes (milligrams of protein in a given plasma pool) by absolute quantification, and metabolic rates by compartmental modeling (Figure 1D). For participants 1 to 12, the HDL proteome was characterized by global proteomics across the 6 HDL sizes (Figure 1D).

### APOA1 and APOA4 levels in plasma, in HDL, and across 6 HDL sizes.

**Figure 2:**
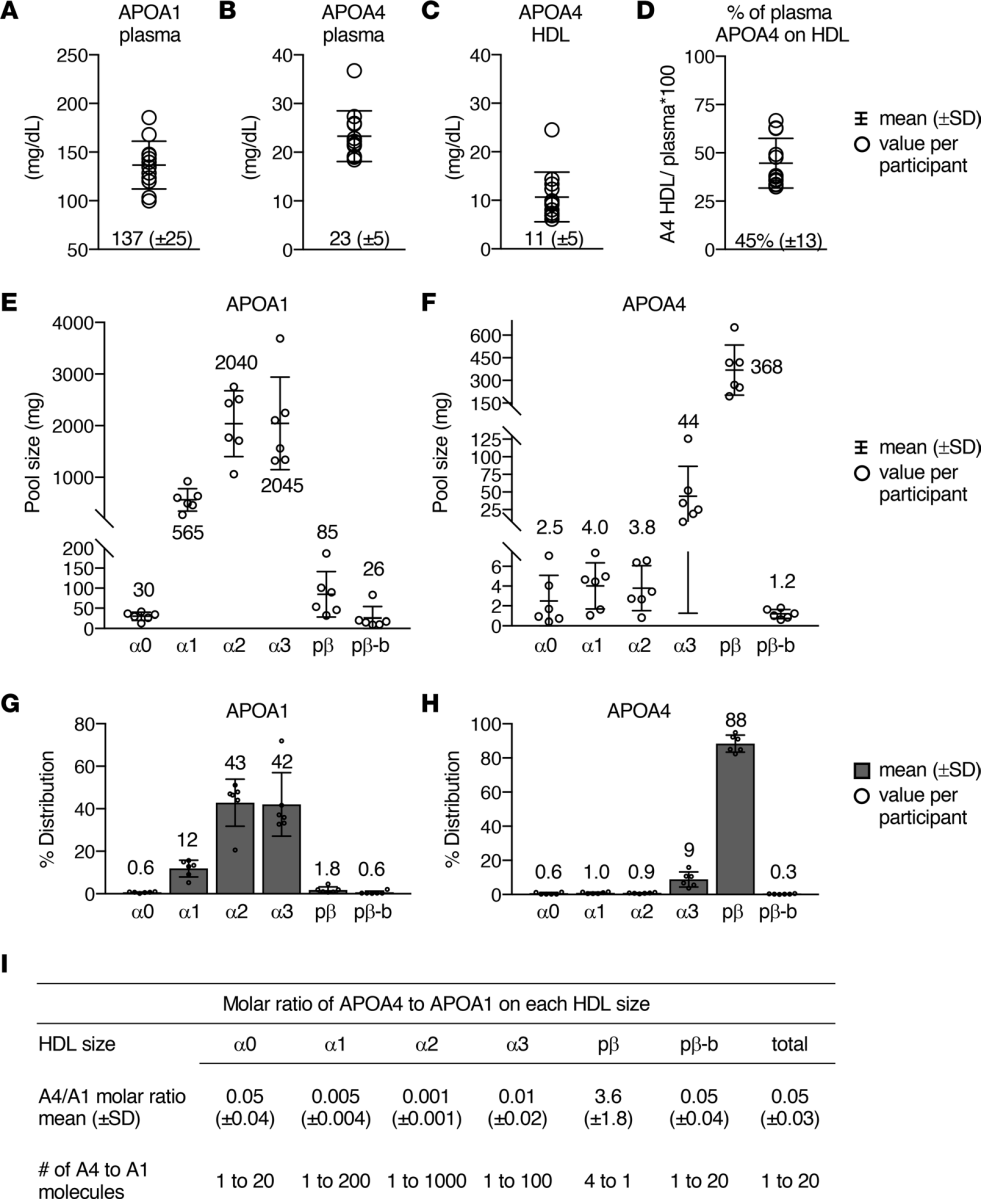
APOA1 and APOA4 levels in plasma, in HDL, and across 6 HDL sizes. Concentration (mg/dL) of APOA1 in plasma (**A**), APOA4 in plasma (**B**), and APOA4 on APOA1-HDL (**C**). Percentage of total plasma APOA4 on HDL (**D**). (**A**–**D**) Bars and the number at the base of each graph are the mean (±SD) for the 12 participants. Each circle represents a value per participant. Pool size (mg) of APOA1 (**E**) and APOA4 (**F**) and percentage distribution of APOA1 (**G**) and APOA4 (**H**) across the 6 HDL sizes in participants 1 to 6. (**E**–**H**) Number above or below each HDL size represents the average pool size (**E** and **F**) or percentage distribution (**G** and **H**) in that size fraction. Bars represent mean (±SD). (**I**) The molar ratio of APOA4 to APOA1 on each HDL size was calculated using the pool size data in **E** and **F**.

We first determined the concentration of APOA1 and APOA4 in plasma in the 12 participants by enzyme-linked immunosorbent assay (ELISA). Average (±SD) levels of plasma APOA1 and APOA4 for the 12 participants were 137 (±25) mg/dL and 23 (±5) mg/dL, respectively (Figure 2, A and B, respectively). These protein concentrations are consistent with previous reports in participants with low HDL-cholesterol and who are overweight or obese (49, 50, 52). We subsequently isolated APOA1-containing particles and determined the amount of APOA4 in this APOA1-HDL. On average, 11 (±5) mg/dL (Figure 2C), or 45% (±13%) (Figure 2D), of total plasma APOA4 was found on APOA1-HDL.

We then used these concentrations of APOA1 and APOA4 on HDL as well as absolute quantification to determine the pool size of APOA1 and APOA4 across the 6 HDL sizes in 6 participants (participants 1 to 6). These pool size values were also used for compartmental modeling described below. Consistent with previous reports, the average pool sizes of APOA1 in alpha0, 1, 2, 3, prebeta, and prebeta-b were 30, 565, 2,040, 2,045, 85, and 26 mg (Figure 2E) (7, 52). Relative to APOA1, APOA4 levels in these sizes were approximately 4- to 1,000-fold lower, at 2.5, 4.0, 3.8, 44, 368, and 1.2 mg, respectively (Figure 2F). These pool size values were then converted to percentage distribution across the 6 HDL sizes. The majority of APOA1 was detected in alpha2 and alpha3 (43% and 42%, respectively) while only 1.8% and 0.6% of APOA1 on HDL were detected in prebeta and prebeta-b, respectively (Figure 2G). Remarkably, the majority of APOA4 was detected in prebeta (88%), with 9% in alpha3, and 1% or less in alpha0, 1, and 2 and prebeta-b (Figure 2H).

We next used the pool size values to determine the molar ratio of APOA4 to APOA1 in each HDL size and in the total HDL fraction. Prebeta was the only fraction in which APOA4 was more abundant than that of APOA1, at 4 molecules of APOA4 to 1 APOA1 (Figure 2I). In the other sizes and in total HDL, 1 molecule of APOA4 was present for every 20 molecules of APOA1 in alpha0, prebeta-b, and total HDL and for every 100, 1,000, and 200 APOA1 molecules in alpha3, alpha2, and alpha1, respectively (Figure 2I).

### APOA4 metabolism across 6 HDL sizes.

**Figure 3:**
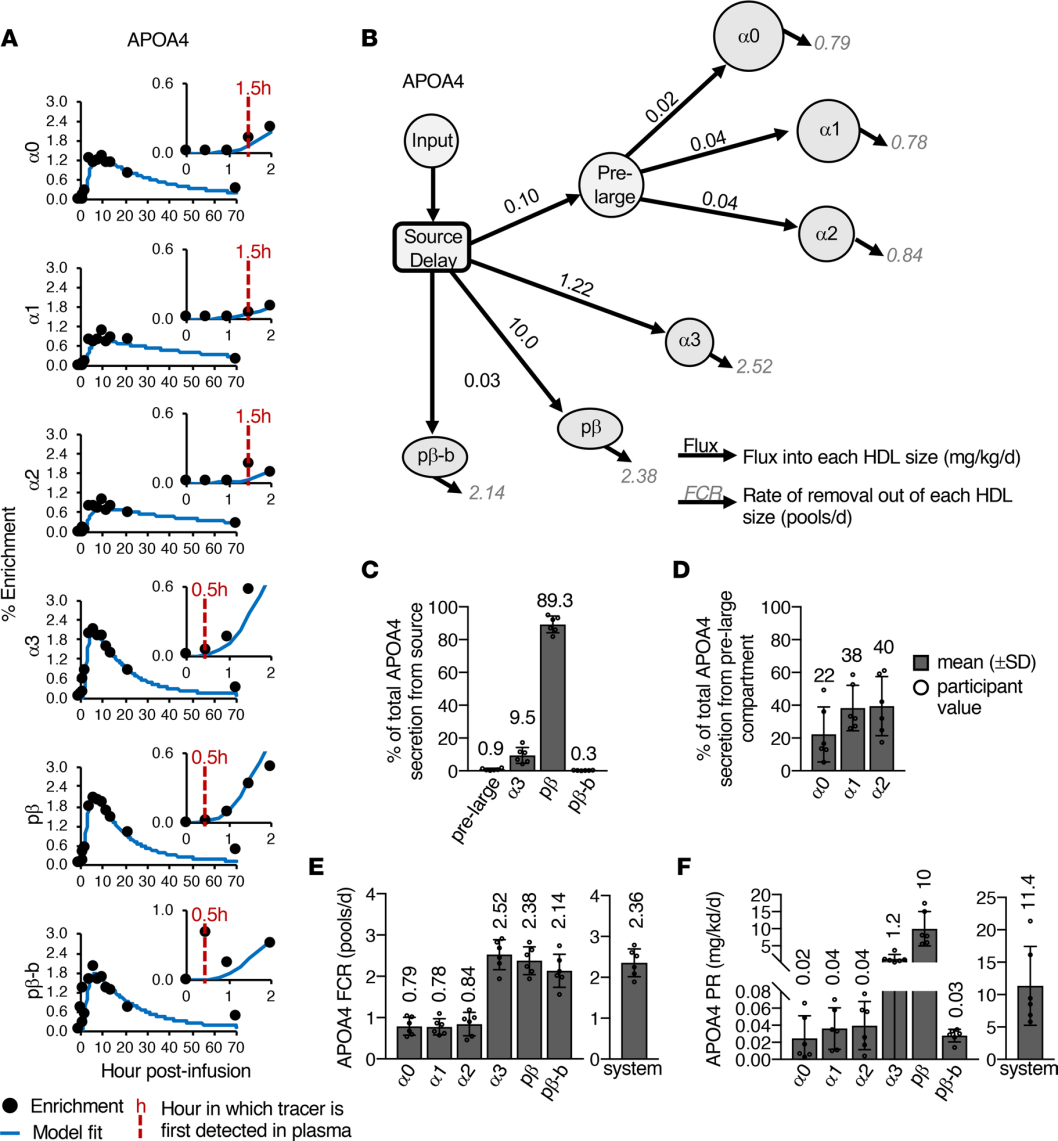
APOA4 metabolism across 6 HDL sizes. (**A**) Representative APOA4 enrichment curves and model fits across 6 HDL sizes (participant 4). Inset shows zoom of the first 2 hours of the time course. Red dashed lines and hour indicate the time of APOA4 tracer appearance in plasma for each size fraction. APOA4 appears by 30 minutes in the small sizes (alpha3, prebeta, prebeta-b) but not until 1.5 hours for the larger sizes (alpha0, 1, 2) in participant 4. (**B**) APOA4 compartmental model used to determine the fractional catabolic rate (FCR; pools/d) and production rate (PR; mg/kg/d) of APOA4 on each HDL size in participants 1 to 6. No size interconversion pathways were detected; thus, only 1 flux pathway (mg/kg/d) into and 1 removal pathway (pools/d) out of each HDL size was detected per HDL size. Flux and FCR values next to arrows represent average values for the 6 participants. (**C**) Percentage of total APOA4 secreted out of the source into the pre-large compartment, alpha3, prebeta, and prebeta-b. (**D**) Percentage of total APOA4 secretion out of the pre-large compartment into alpha0, 1, and 2. APOA4 FCR (**E**) and PR (**F**) on each HDL size and in the total model system. The total model system FCR and PR represent the rate of total APOA4 being removed and the total amount of APOA4 entering the HDL system per day, respectively. (**C**–**F**) Bar height and error bars represent the mean (±SD) of the 6 participants.

The APOA4 tracer enrichment curves across the 6 HDL sizes were determined by targeted mass spectrometry (using the peptides listed in Supplemental Table 2) and found to be strikingly different across the HDL sizes (Figure 3A, Supplemental Figure 1A, and Supplemental Table 3). APOA4 on the small HDL sizes appeared in circulation by 30 minutes (Figure 3A, inset; Supplemental Figure 1B) and peaked at a median time of 6 hours with a median peak enrichment of approximately 2.0% (Figure 3A and Supplemental Figure 1A). In contrast, APOA4 enrichment in the larger sizes appeared in circulation later — between 1 and 2 hours — peaked at a median of 6 hours, and reached a peak enrichment half that of the smaller sizes (median 1.0%–1.2%) (Figure 3A and Supplemental Figure 1, A and B). These distinct enrichment curve patterns indicate that APOA4 on small and large HDL each has a distinct origin and unique metabolism and possibly different functions.

We subsequently used compartmental modeling to determine the metabolism of APOA4 across the 6 HDL sizes in circulation. The APOA4 model contained an input, a source delay compartment, and 6 compartments representing each HDL size (Figure 3B). An additional compartment was needed between the source and the large HDL size compartments in order to account for the delayed appearance of APOA4 on large, relative to small, HDL sizes (Figure 3B). We speculate that this additional intermediary compartment (which we term “pre-large” in Figure 3) may be physiologically representative of chylomicrons and may account for the time it takes for APOA4 to transfer from chylomicrons to HDL in circulation. The majority of APOA4 secretion from the source compartment was into prebeta (89.3%), with smaller amounts into alpha3 (9.5%), prebeta-b (0.3%), and the pre-large compartment (0.9%) (Figure 3, B and C). Similar amounts of APOA4 were secreted from the pre-large compartment into each of the large HDL sizes (Figure 3, B and D). No size interconversion was detected; all APOA4 entering a given size fraction was subsequently removed from that size.

Given that the general enrichment curve shape was similar among the large and among the small HDL sizes, the FCRs across the large and across the small sizes were also similar. The average APOA4 FCR for large HDL was between 0.78–0.84 pool/d, while the APOA4 FCR on the small sizes was about 3 times faster at 2.1–2.5 pools/d (Figure 3E and Supplemental Table 4). When considering all HDL sizes, the total rate of APOA4 catabolism out of the entire modeled system was similar to that of prebeta, at 2.36 pools/d (Figure 3E, system; Supplemental Table 4). The production rate of APOA4 into the large sizes and into prebeta-b ranged between 0.02 to 0.04 mg/kg/d and was almost 100-fold higher for alpha3 and 1,000-fold higher for prebeta (Figure 3F and Supplemental Table 4). Overall, a total of 11.4 mg/kg of APOA4 entered the HDL system per day (Figure 3F, system; Supplemental Table 4).

The APOA4 kinetic parameters (FCR, PR, and pool size) were then compared between the 3 female and 3 male participants included in the kinetic analysis. The APOA4 kinetic parameters did not differ between women and men in any of the HDL sizes (Supplemental Table 4). This lack of sex differences is consistent with a previous study showing that APOA4 levels in plasma and APOA4 kinetic parameters on triglyceride-rich lipoproteins are similar between females and males (50).

### Comparison of APOA4 and APOA1 enrichment curves.

**Figure 4:**
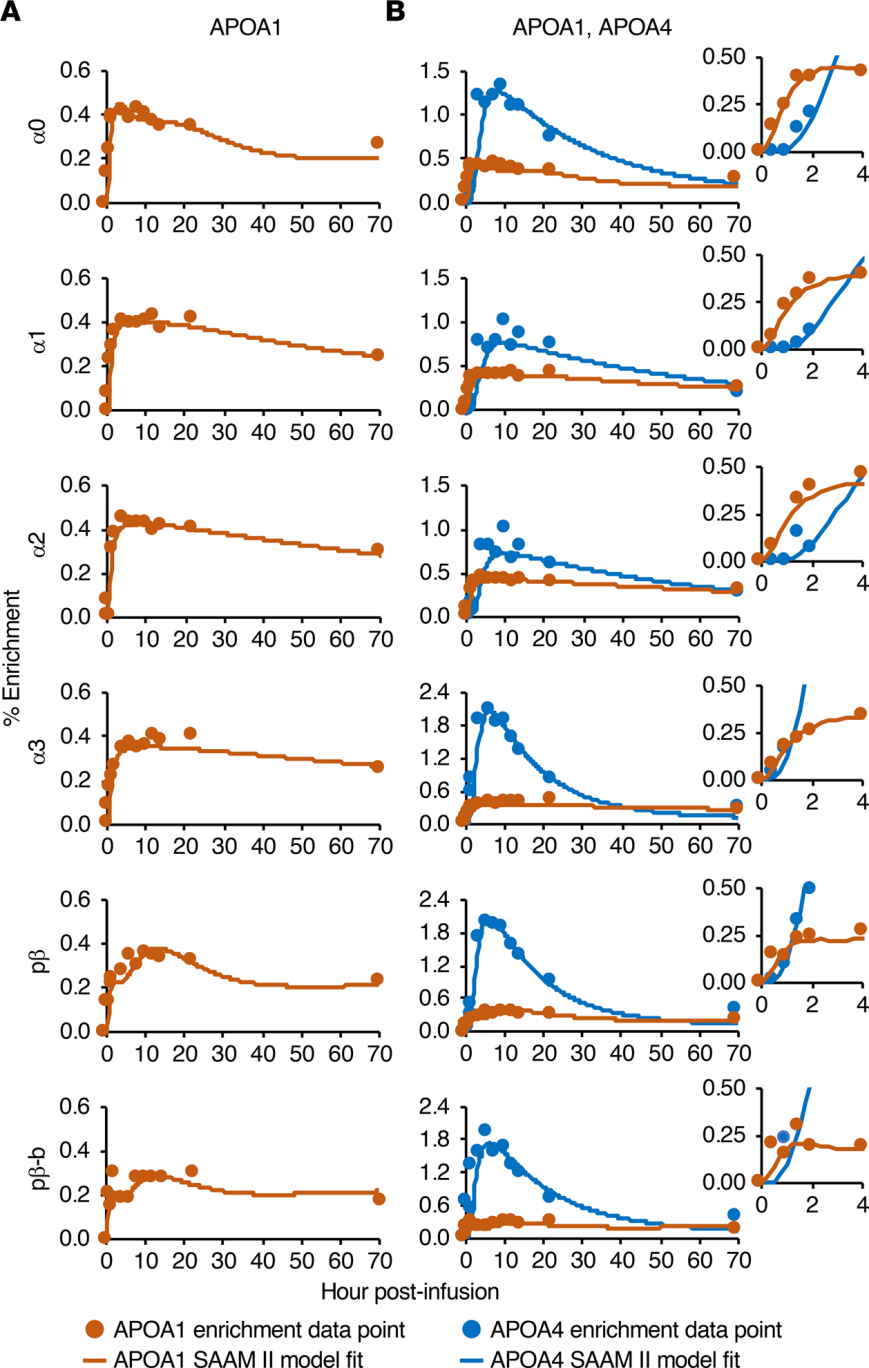
APOA1 and APOA4 enrichment curve comparison across 6 HDL sizes. (**A**) Representative APOA1 enrichment curves and model fits across 6 HDL sizes (participant 4). (**B**) Direct comparison of APOA1 and APOA4 enrichment curves across the size fractions (participant 4). The inset shows a zoom of the first 4 hours of the time course.

APOA1 tracer enrichment across the 6 HDL sizes was also determined by targeted mass spectrometry (using the peptides listed in Supplemental Table 2) and compared with that of APOA4 (Figure 4A, Supplemental Figure 2A, and Supplemental Table 3). Unlike APOA4, the APOA1 enrichment curve ascending and descending slopes were more gradual across the sizes and only reached a median peak enrichment of 0.4%–0.6% (Figure 4B and Supplemental Figure 2A). The time of APOA1 peak enrichment was also more variable across the sizes and participants, with a median of 4 to 18 hours (Figure 4A and Supplemental Figure 2A). APOA1 on all HDL sizes appeared in circulation by 30 minutes (Figure 4A and Supplemental Figure 2A). Interestingly, when looking at the first 2 hours of the time course, the ascending slope of APOA1 crossed the origin, while the ascending slope of APOA4 was slightly delayed and shifted to the right, even in the smaller sizes in which APOA4 tracer was also detected by 30 minutes (Figure 4B, inset). The differences in these early ascending slopes likely reflect organ-specific differences in production and secretion between liver-derived APOA1 and small intestine–derived APOA4. Additionally, the delay in APOA4 may reflect the time necessary for small intestine–derived particles to travel through the lymph before entering circulation.

The enrichment curves for APOA1 on prebeta and prebeta-b were also compared. APOA1 on prebeta and prebeta-b tended to have a similar shape, but the total enrichment level for prebeta-b was lower for most participants (Figure 4A and Supplemental Figure 2B). The similar enrichment curves shapes suggest that prebeta and prebeta-b have a similar metabolism and possibly a similar origin.

### APOA1 metabolism across 6 HDL sizes.

**Figure 5:**
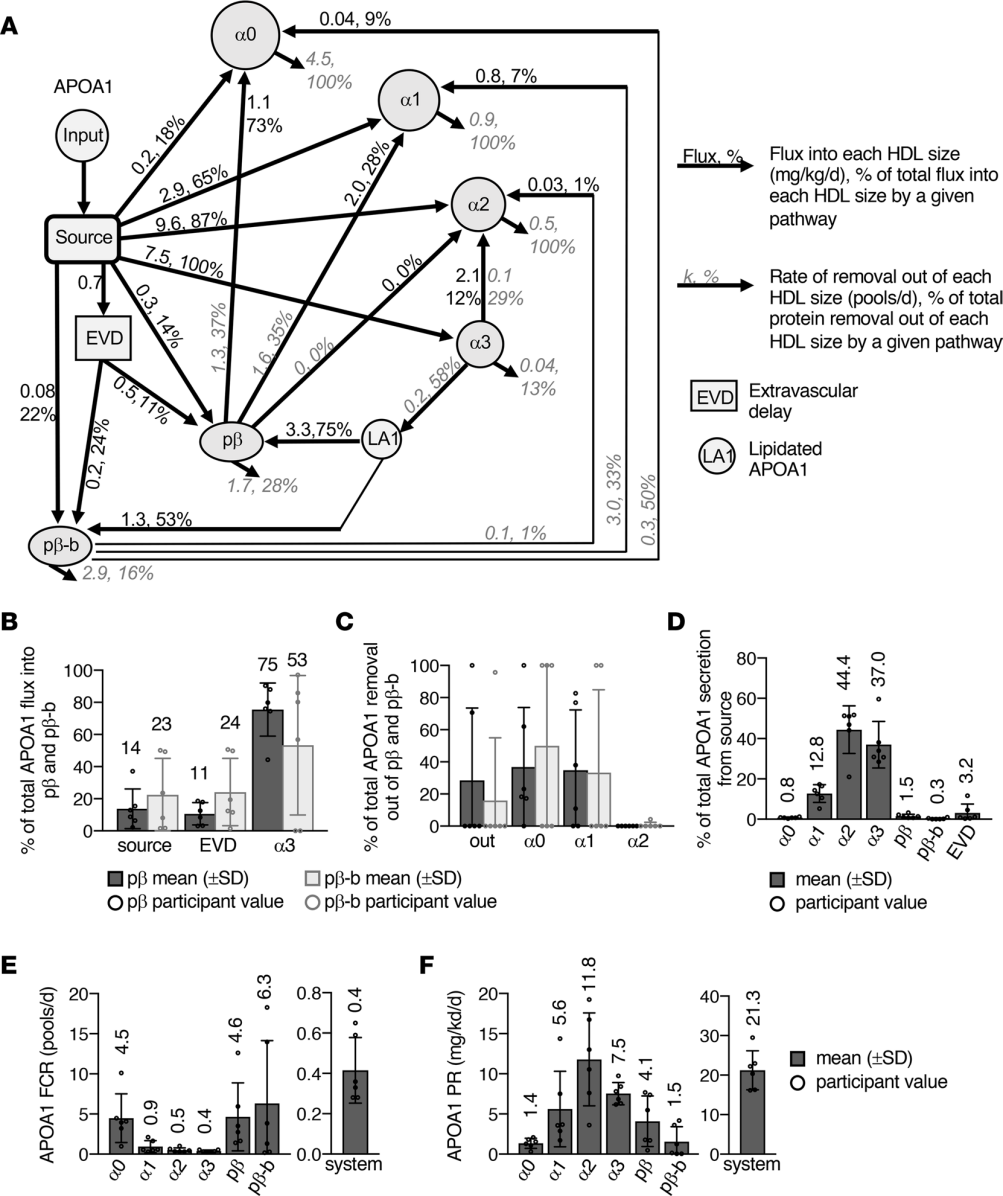
APOA1 metabolism across 6 HDL sizes. (**A**) APOA1 compartmental model used to determine the FCR (pools/d) and PR (mg/kg/d) of APOA1 on each HDL size. Numbers next to arrows represent average flux (mg/kg/d), percentage of total flux, rate of removal (pools/d), and percentage of total protein removal values for participants 1 to 6. (**B**) Percentage of total APOA1 flux into prebeta and into prebeta-b from the source, delay, and alpha3 compartments. (**C**) Percentage of total APOA1 removal out of prebeta and out of prebeta-b by each removal pathway (out of the model system, alpha0, alpha1, and alpha2). (**D**) Percentage of total APOA1 secretion (mg/kg/d) from the source compartment and into alpha0, 1, 2, and 3; prebeta; prebeta-b, and the extravascular delay. APOA1 FCR (**E**) and PR (**F**) on each HDL size and in the total model system. The total model system FCR and PR represent the rate of total APOA1 being removed and the total amount of APOA1 entering the HDL system per day, respectively. (**B**–**F**) Bar height and error bars represent the mean (±SD) of the 6 participants.

We next used compartmental modeling to determine the metabolism of APOA1 across the 6 HDL sizes and compared it with that of APOA4. The APOA1 kinetic model developed previously (7, 8, 52) was expanded upon to include prebeta-b (Figure 5A). The APOA1 model included an input, a source, 6 HDL size compartments, an extravascular delay, and a lipidated APOA1 compartment (see Methods, “Compartmental modeling”) (Figure 5A). The addition of APOA1 prebeta-b did not change the metabolic structure of the APOA1-HDL system, and the pathways needed to describe the metabolism of APOA1 prebeta-b were the same as those for prebeta (Figure 5A). The majority of prebeta and prebeta-b both originated from alpha3 (75% and 53%, respectively), with smaller amounts from the source and extravascular delay compartments (Figure 5B). Removal pathways out of the model system and size expansion pathways into alpha0 and alpha1 were also detected for both prebeta and prebeta-b, though the detection and percentage of total APOA1 removal by a specific removal pathway tended to vary across participants (Figure 5C). Unlike APOA4, only 5% of total APOA1 was secreted into the prebeta system, either directly into prebeta (1.5%) and prebeta-b (0.3%) or indirectly via the extravascular delay (3.2%) (Figure 5D). The FCR of APOA1 on prebeta-b (6.3 pools/d) was similar to that of prebeta (4.6 pools/d) (Figure 5E) and 2 to 3 times faster than that of APOA4 on these sizes (Supplemental Table 4). The PR of APOA1 prebeta-b was a third that of prebeta (Figure 5F and Supplemental Table 4). In addition to APOA1 on prebeta and prebeta-b, the APOA1 metabolic parameters in the alpha sizes were consistent with previous reports (7, 8, 52).

### Characterization of the proteomes of prebeta-b and other HDL sizes.

Since we identified both APOA4 and APOA1 in prebeta-b, we wanted to determine what other proteins reside in this fraction, as at least some of these proteins may be components of small intestine–derived APOA4-HDL. The proteome of the 5 large HDL sizes was also determined and compared with that of prebeta-b (a detailed analysis of the 5 larger sizes has been described previously; refs. 7, 52).

**Figure 6:**
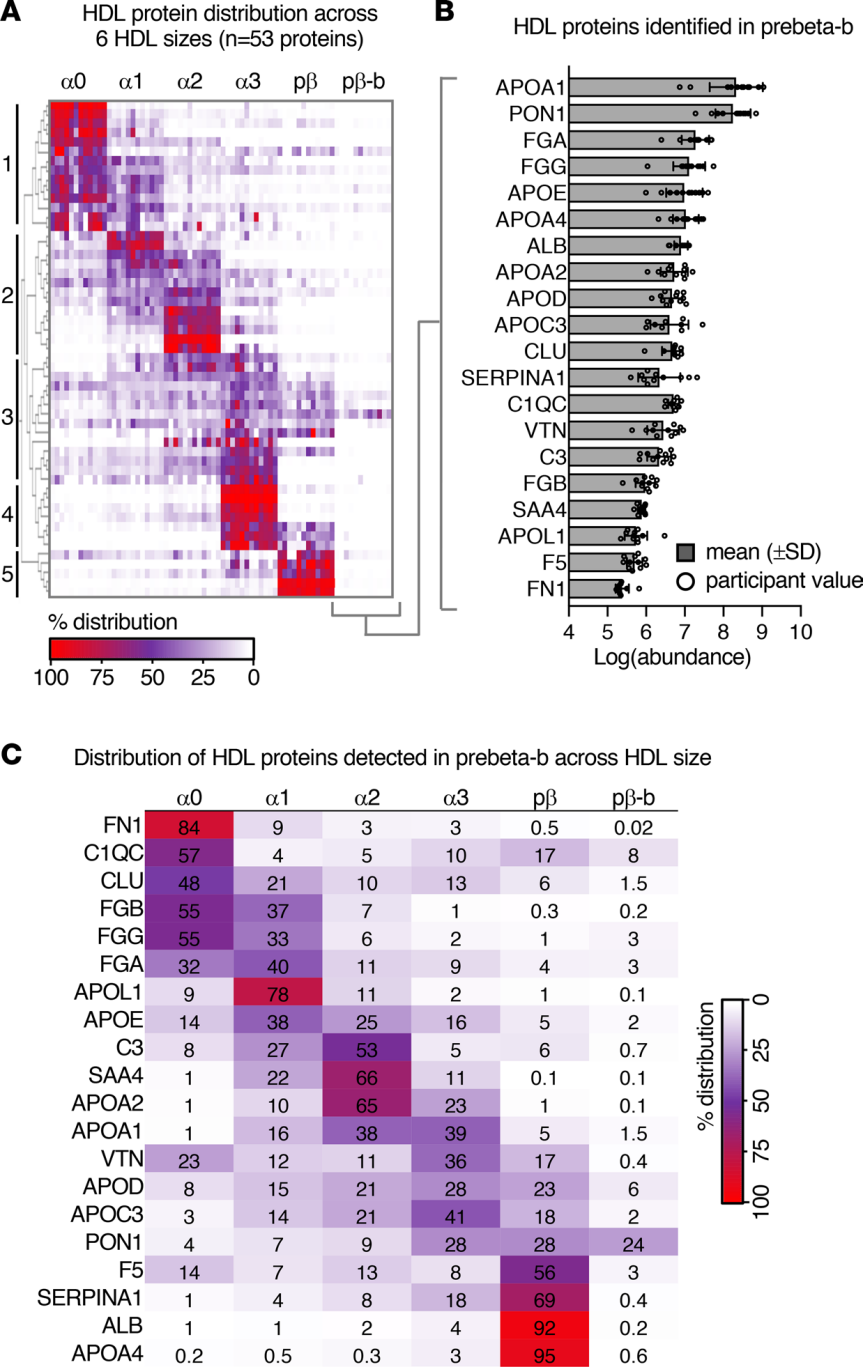
Characterization of the prebeta-b proteome. (**A**) Relative percentage distribution of the 53 HDL proteins detected in 12 participants across 6 HDL sizes. Numbers 1 to 5 indicate the 5 size-specific protein clusters (1, proteins that dominate in alpha0; 2, alpha1 and/or alpha2; 3, alpha2 and alpha3 or alpha3 and prebeta; 4, alpha3; 5, prebeta). No protein cluster specific to prebeta-b was identified. The relative percentage distribution of each protein in each HDL size was determined by taking each protein’s abundance value in a given size divided by the total abundance for that protein in the 6 HDL sizes per participant. (**B**) Log protein abundance values for the 20 proteins identified in prebeta-b in ≥75% (*n* = 9 to 12) of participants. Bar height and error bars represent the mean (±SD). (**C**) Average percentage distribution of the 20 prebeta-b proteins across the 6 HDL sizes in 9 to 12 participants. PON1, serum paraoxonase 1; FGA, fibrinogen alpha chain; FGG, fibrinogen gamma chain; APOE, apolipoprotein E; ALB, albumin; APOA2, apolipoprotein A2; APOD, apolipoprotein D; APOC3, apolipoprotein C3; CLU, clusterin; SERPINA1, alpha-1-antitrypsin; C1QC, complement C1q subcomponent subunit C; VTN, vitronectin; C3, complement C3; FGB, fibrinogen beta chain; SAA4, serum amyloid A-4; APOL1, apolipoprotein L1; F5, coagulation factor V; FN1, fibronectin.

The proteome of each HDL size was determined by global proteomics for participants 1 to 12. The abundance value for each identified protein was determined by first taking the area under the curve of the extracted ion chromatograms of a given protein’s peptides, then taking the sum of these peptide quantification values (Proteome Discoverer manual, Quantification Methodologies section). The Proteome Discoverer exported protein abundance data for each participant are shown in Supplemental Table 5, and the peptides identified for each protein are listed in Supplemental Table 6. We then identified 53 HDL proteins that were present in 1 or more size fractions in all 12 participants (Supplemental Table 7). Each protein’s relative distribution across the 6 HDL sizes was then determined by taking each protein’s abundance value in a given HDL size divided by the total abundance for that protein across the 6 HDL sizes per participant. The protein distribution profiles were then clustered based on their relative distribution patterns across the HDL sizes. Five distinct, size-specific clusters were formed: proteins enriched in (cluster 1) alpha0, (cluster 2) alpha1 and/or alpha2, (cluster 3) alpha2 and alpha3 or alpha3 and prebeta, (cluster 4) alpha3, and (cluster 5) prebeta (Figure 6A and Supplemental Table 7). Interestingly, for clusters 1 to 4, proteins with similar functions tended to reside in the same cluster. For instance, cluster 1 contained several complement proteins and immunoglobulins, suggesting that alpha0 may play a role in the immune response. Additionally, clusters 2 and 3 contained several proteins known to regulate cholesterol metabolism (APOA1, APOA2, APOC3, APOE) while cluster 4 contained several proteins with protease inhibition function, including alpha-1-antichymotrypsin (SERPINA3) and antithrombin-III (SERPINC1). These results suggest that various HDL functions may localize to specific size ranges.

Unlike the larger sizes, no proteins dominated in prebeta-b, and therefore, no protein cluster specific to prebeta-b was identified (Figure 6A and Supplemental Table 7). However, 34 proteins were still detected in prebeta-b in at least 1 participant (Supplemental Table 7), and 20 of these proteins were identified in prebeta-b in the majority (≥75%) of participants (Figure 6B and Supplemental Table 7). The abundance values for these 20 prebeta-b proteins were compared (Figure 6B): the APOA1 signal was the most abundant in prebeta-b, followed by serum PON1, FGA, FGG, APOE, APOA4, ALB, APOD, APOC3, CLU, and SERPINA1 (Figure 6B). In addition to APOA4, PON1, APOE, APOD, APOC3, and SERPINA1 have all been shown to be produced by the small intestine (24, 53–55), suggesting that these proteins may reside on small intestine–derived HDL.

We then took a closer look at the relative distribution of these 20 prebeta-b proteins across the 6 HDL sizes to determine how much of each protein resides in prebeta-b relative to total HDL (sum of the 6 HDL sizes) (Figure 6C). About a quarter (24%) of total HDL PON1 was detected in prebeta-b, followed by 8% of CIQC; 6% of APOD; 3% of FGG, FGA, and F5; 2% of APOE and APOC3; and 1.5% of CLU and APOA1 (Figure 6C). Less than 1% of total HDL APOA4, and the remaining prebeta-b proteins, were found in prebeta-b (Figure 6C).

## Discussion

**Figure 7:**
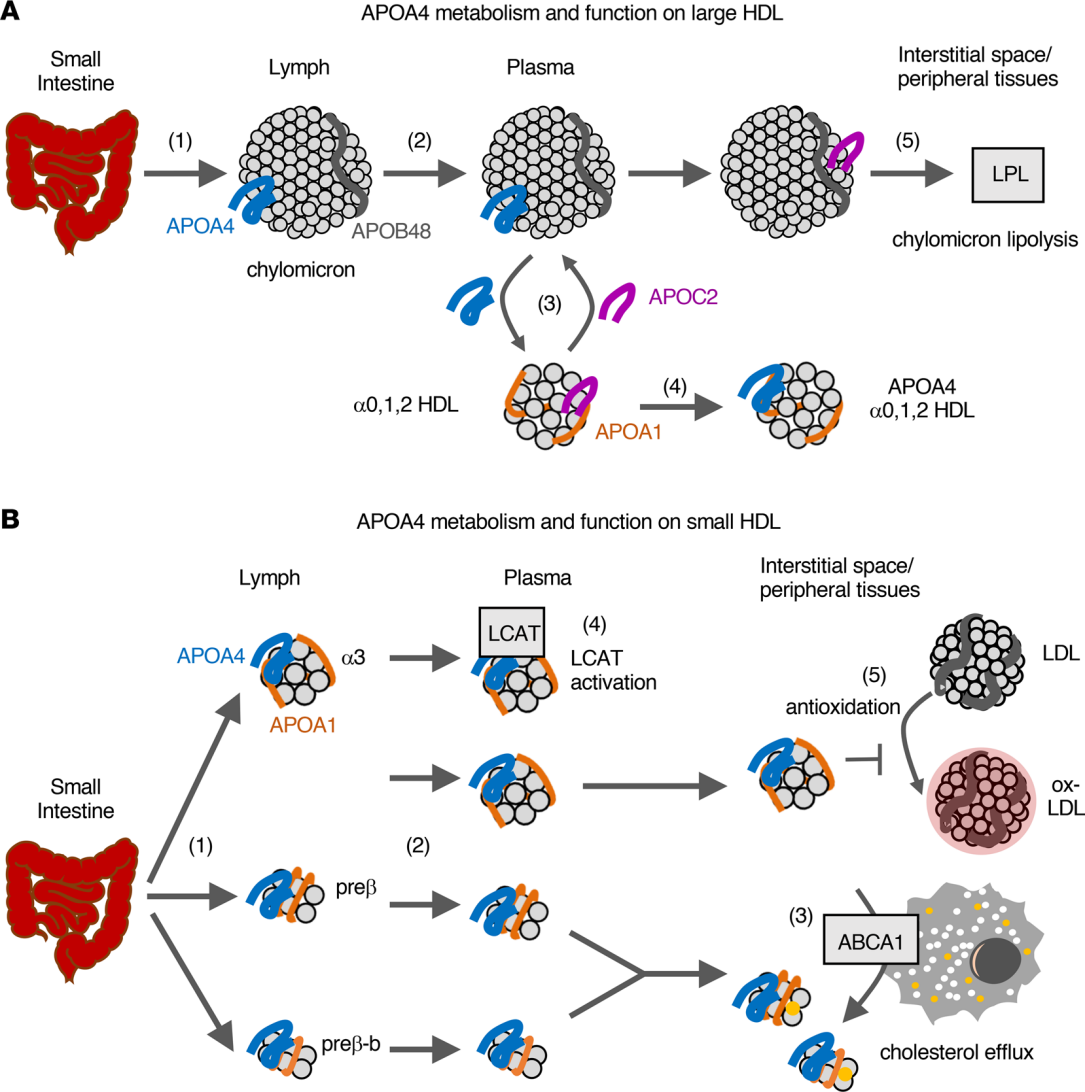
Model of APOA4 metabolism, origin, and function on large and small HDL. (**A**) Model of APOA4 metabolism on large HDL. APOA4 is synthesized and secreted into the lymph on the surface of chylomicrons (step 1). The chylomicrons with APOA4 then enter circulation (step 2), where APOA4 is exchanged for APOC2 on liver-derived large HDL (step 3), generating APOA4-containing large HDL (step 4). Once on chylomicrons, APOC2 activates triglyceride lipolysis and uptake via lipoprotein lipase (LPL) on peripheral tissues (step 5). (**B**) Model of APOA4 metabolism on small HDL. The small intestine synthesizes and secretes small HDL with APOA4 into the lymphatics (step 1), and these HDL then enter plasma circulation (step 2). APOA4 on prebeta and prebeta-b particles efflux cholesterol from peripheral tissues via ATP-binding cassette transporter A1 (ABCA1) (step 3). APOA4 on alpha3 HDL in plasma activates lecithin-cholesterol acyltransferase (LCAT) (step 4) and the same or a distinct alpha3 subspecies functions to protect LDL from becoming oxidized (ox-LDL) (step 5).

This study presents a comprehensive picture of the metabolism of APOA4 and its relationship to the major HDL protein APOA1 across 6 HDL sizes in humans. The metabolic structures of APOA4 on large (alpha0, 1, and 2) and small (alpha3, prebeta, prebeta-b) HDL are distinct. APOA4’s appearance on large HDL in circulation is delayed (1–2 hours postinfusion), while APOA4 on small HDL appears rapidly (by 30 minutes). Additionally, once in circulation, APOA4 on small HDL is catabolized almost 3 times faster than APOA4 on large HDL. These distinct metabolic structures of APOA4 on large and small HDL indicate that each group of particles likely have a unique origin and function. We hypothesize that APOA4 on large HDL originates from chylomicron transfer and functions to promote chylomicron lipolysis, while APOA4 on small HDL originates directly from the small intestine and activates cholesterol efflux and cholesterol esterification, and potentially a range of additional functions, as discussed in detail below and summarized in Figure 7.

Due to the delayed appearance of APOA4 on large HDL, an additional compartment was needed in the APOA4 metabolic model between the source compartment, representative of the small intestine, and the 3 large HDL size plasma compartments (Figure 3B). We speculate that this additional compartment is physiologically representative of chylomicrons (Figure 7A). APOA4 is synthesized by the small intestine, incorporated onto the surface of chylomicrons, and then secreted into the lymphatics (Figure 7A, step 1). Chylomicrons then travel through the lymph and enter systemic circulation via the thoracic duct (Figure 7A, step 2). Once in circulation, APOA4 detaches from the chylomicron surface and transfers to, but not from, HDL, as has been shown in humans using radiolabeled APOA4 injected into plasma on the surface of chylomicrons (30, 32). In vitro studies suggest that this APOA4 on chylomicrons is transferred to HDL in exchange for APOC2 (Figure 7A, step 3) (33, 34), resulting in the generation of APOA4-containing HDL (Figure 7A, step 4). Once on chylomicrons, APOC2 can bind to and activate LPL, thus facilitating triglyceride lipolysis and uptake by peripheral tissues (Figure 7A, step 5). In vivo studies in mice also support a role for APOA4 in mediating chylomicron lipolysis, as chylomicrons from APOA4-deficient mice are metabolized and cleared from plasma more slowly compared with chylomicrons from wild-type mice (35). Overall, this mechanism of APOA4 and APOC2 exchange between chylomicrons and HDL may account for the generation and delayed appearance of APOA4 on large HDL in plasma in humans in vivo.

Consistent with these in vitro and mouse studies, several additional studies in humans also support the notion that APOA4 on large HDL is the result of transfer from chylomicrons. First, APOC2 is primarily found on large HDL in human plasma, suggesting that large HDL is likely involved in the APOC2 and APOA4 transfer in humans (10). Also, in patients with abetalipoproteinemia, a genetic disorder in which chylomicrons and other APOB lipoproteins are not synthesized, APOA4 is still detected on small HDL, but not on large HDL, suggesting that the presence of APOA4 on large HDL may be dependent on chylomicron production (56). Furthermore, if APOA4 on chylomicrons does indeed transfer to HDL, we would expect APOA4 on chylomicrons to appear in circulation earlier and peak earlier than that of APOA4 on large HDL. Although we did not monitor chylomicron APOA4 metabolism in the present study, a previous kinetic study showed that APOA4 on triglyceride-rich lipoproteins (which includes chylomicrons, VLDL, and remnants) appeared in circulation by 1 hour (earlier time points were not monitored) and was metabolized almost 40% faster than that of APOA4 in plasma (50). With its early appearance and rapid metabolism, APOA4 on chylomicrons may serve as the precursor for the later appearing, and more slowly metabolized, APOA4 on large HDL. Last, an average of 3% of total plasma APOA4 has been identified on chylomicrons (30, 50, 56). We also found that about 3% of APOA4 on HDL resides on the large HDL sizes (Figure 2H), further supporting the notion that APOA4 on large HDL originates from chylomicron transfer. Taken together, these data strongly suggest that APOA4 on large HDL may indeed originate from chylomicrons.

APOA4 on each small HDL size was secreted directly from the source compartment, representative of the small intestine, and remained on that given size until removed from circulation (Figure 3B). Similar to APOA4 on chylomicrons, we hypothesize that these small APOA4 HDL particles are synthesized directly by the small intestine, are secreted into the lymphatics (Figure 7B, step 1), and enter plasma circulation via the thoracic duct (Figure 7B, step 2). Direct measurement of small intestine–derived HDL in the lymph is challenging, especially in humans, given that liver-derived HDL from plasma can also enter lymphatic circulation. However, evidence in the human intestinal Caco-2 cell line and in a rodent small intestine in situ perfusion model has shown that APOA4-containing particles in the small alpha3 and prebeta size range are synthesized directly by the small intestine (28, 29) and secreted into circulation via the lymphatics (29). Intestine-derived HDL are small discoidal and small spherical protein- and phospholipid-rich particles that contain APOA4 and APOA1, while liver-derived HDL tend to be larger and spherical and contain more cholesterol ester (15, 28, 29). The molar ratio of APOA4 to APOA1 produced in the Caco-2 culture media was 2.74 APOA4 to APOA1 (28), within range of our average (±SD) APOA4 to APOA1 molar ratio in prebeta (3.6 [±1.8]), further supporting the notion that these particles are likely synthesized and secreted by the small intestine in humans in vivo.

In addition to activating chylomicron lipolysis, APOA4 has been implicated in a large range of functions, several of which are likely orchestrated by small APOA4 HDL. APOA4 promotes HDL-mediated reverse cholesterol transport by activating cholesterol efflux via ABCA1 (36, 37) and cholesterol esterification via LCAT (38), as shown in vitro. Also, APOA4 functions as an antioxidant and protects LDL and fasting lymph lipoproteins from copper-induced and macrophage-induced oxidation, respectively, in vitro (39). These roles of APOA4 in promoting reverse cholesterol transport and protecting LDL from oxidation may at least in part explain why human APOA4 transgenic mice have significantly reduced atherosclerosis compared with their wild-type counterparts (57). Given that prebeta-sized HDL, not spherical alpha particles, activate ABCA1, and that the majority of APOA4 resides on prebeta, it is likely that APOA4 on prebeta HDL is the primary APOA4 subspecies responsible for mediating ABCA1 cholesterol efflux. Due to its low abundance, APOA4 on prebeta-b likely does not contribute substantially to cholesterol efflux (Figure 7B, step 3). Furthermore, APOA4 on alpha3 is likely the APOA4 subspecies that activates LCAT, as LCAT primarily resides on this HDL size in human plasma (51, 52) (Figure 7B, step 4). Last, APOA4 on alpha3 may also be the APOA4 subspecies that protects LDL from oxidation, as alpha3 particles have greater antioxidant function compared with larger HDL (10) (Figure 7B, step 5).

Our APOA4 kinetic findings are consistent with previous studies of APOA4 metabolism on HDL. In addition to our preliminary kinetic study, which analyzed the metabolism of APOA4 on alpha3 and prebeta HDL (7), only 2 previous studies have analyzed APOA4 metabolism in HDL (30, 32). Using exogenous radiolabeled APOA4, these studies found that the FCR of APOA4 on HDL was 0.62 (30) and 0.70 pool/d (32), similar to what we found for APOA4 on large HDL (0.78–0.84 pools/d) (Figure 3E). Additionally, APOA4 catabolism in the density >1.21 g/mL fraction, which has been shown to contain prebeta HDL (58), was 3 times faster, at 1.81 pools/d, than that of APOA4 on HDL (30), similar to the fold difference we found between APOA4 on large and small HDL (Figure 3E).

In humans, APOA1 metabolism across multiple HDL sizes showed that HDL is directly secreted into circulation and remains mainly within that given size throughout its time in circulation (7, 8, 52). In these studies, prebeta was derived primarily from alpha3 particle remodeling and was not found to be a primordial HDL particle that sequentially grows in size as it collects cholesterol (7, 8, 52). In the present study, we also determined the metabolism of APOA1 on a smaller prebeta particle, prebeta-b. We found that the metabolism of APOA1 on prebeta-b was similar to that of prebeta: the majority of APOA1 in both prebeta and prebeta-b originated from alpha3, with smaller amounts from the source and the extravascular delay processing compartments; and both were active in size expansion, as flux pathways into alpha0 and alpha1 were detected from both sizes (Figure 5). The only marked metabolic difference between the 2 particles was that the pool size of APOA1 on prebeta-b (26 mg) was about a third that of prebeta (85 mg) (Figure 2E).

Along with characterizing the metabolism of APOA1 on prebeta-b, we also determined its proteome in order to gain better insight into its composition and potential relationship to small intestine–derived APOA4 HDL. Unlike the larger sizes, prebeta-b did not contain any proteins that were enriched in this size fraction (Figure 6A). This result may not be surprising given that the low abundance (<1% of total APOA1) and small size of prebeta-b may not make it conducive to carrying a large number of additional proteins. However, in addition to APOA4, several proteins were still identified in this fraction, such as a quarter of total HDL PON1 and a small amount of a few other HDL proteins, such as APOC3, APOD, APOE, and SERPINA1. Interestingly, as with APOA4, many of these proteins can be made by the small intestine (24, 53–55) and are enriched in APOA4 HDL (12), indicating that several of these prebeta-b proteins may reside on APOA4-containing, small intestine–derived HDL.

This study has several limitations. First, our study is limited to participants with low HDL-cholesterol and who are overweight or obese and does not contain a normal HDL-cholesterol, lean comparison group. It is not yet known whether this size-specific APOA4 metabolism is also observed in healthy individuals. However, given that the metabolic structure of the APOA1 compartmental model is the same between normal weight, normal HDL-cholesterol and overweight/obese, low HDL-cholesterol individuals (8), we speculate this to be the same for APOA4 on HDL as well. Second, although our compartmental modeling results support the notion that APOA4 on small HDL in humans is directly secreted by the small intestine, we did not directly assess APOA4 small intestine synthesis and secretion. It would be of interest to look at APOA4 synthesis and secretion on HDL using a model system, such as human small intestinal organoids, to directly assess APOA4 HDL secretion by the small intestine in humans. Third, we did not directly measure APOA4 metabolism on chylomicrons and thus can only speculate that the pre-large compartment is indeed representative of chylomicrons. Fourth, in addition to small intestine enterocytes, hepatocytes have also been shown to express relatively low amounts of APOA4 mRNA (16, 17). The contribution of the liver to plasma APOA4 is not well understood but has been estimated to be <5% in patients after liver transplant (18). Thus, it is possible that hepatic APOA4 may contribute to the APOA4 HDL monitored in this study.

Last, an additional limitation is that we cannot clearly explain the 4:1 APOA4/APOA1 molar ratio in prebeta on a structural level. In vitro studies have shown that purified, lipid-free APOA4 monomers and dimers are found at ~6.7 nm (~prebeta size) and ~7.9 nm (alpha3 size), respectively, when run on native gels (59). These data suggest that an HDL particle containing more than 2 molecules of APOA4 would likely be larger than prebeta/alpha3. However, a study in human intestinal Caco-2 cells detected prebeta and alpha3 APOA4-containing particles in the culture media, and these particles had a molar ratio of 2.74 APOA4 to APOA1 molecules, within range of our average (±SD) prebeta molar ratio (3.6 [±1.8]). Unlike the purified, lipid-free APOA4 used in APOA4 structure studies, native APOA4-containing HDL particles contain lipid as well as APOA1 and other proteins (12, 32). Thus, it is possible that the addition of lipid and/or APOA1 allows APOA4 particles to accommodate additional proteins while remaining in the prebeta/alpha3 size. A better understanding of how lipids and proteins influence the structure and composition of APOA4-containing particles is needed to better explain our molar ratio data. It is also likely that the 4:1 APOA4/APOA1 molar ratio in prebeta results from different APOA4-HDL subspecies, as several proteins — such as APOC3, APOD, APOE, PON1, and SERPINA1 — are present in APOA4-containing HDL (12).

In closing, we found that APOA4 on HDL is organized into 2 particle systems — large and small HDL — each of which has its own unique metabolism, origin, and function. To date, we have used targeted mass spectrometry to determine the metabolism of 12 HDL proteins across multiple HDL sizes (7, 51, 52). Of these proteins, APOA4 is the first to show such marked differences across the HDL sizes. Given APOA4’s wide range of functions, our findings may serve as the molecular basis for the development of APOA4-targeted therapeutics for diseases such as dyslipidemia, clinical complications of atherosclerosis, diabetes, obesity, Alzheimer’s disease, and other inflammatory conditions.

## Methods

### Participant recruitment

We recruited 12 participants with low HDL-cholesterol (≤55 mg/dL for women, ≤45 mg/dL for men) and who were overweight or obese (body mass index > 25 kg/m^2^) (Supplemental Table 1). Exclusion criteria included high LDL-cholesterol (>190 mg/dL); very low HDL-cholesterol (<20 mg/dL); very high fasting triglycerides (>500 mg/dL); ApoE genotypes E2E2, E2E4, or E4E4; use of medications or therapies that can alter lipid levels; secondary hyperlipidemia; hepatic or renal complications; diabetes mellitus; and pregnancy.

### Metabolic tracer study

The 12 participants consumed a controlled, healthful diet high in unsaturated fat (40% fat [23% monounsaturated, 7% polyunsaturated, 10% saturated], 45% carbohydrate, 15% protein, 180 mg cholesterol) for 4 weeks prior to the kinetic study. The controlled diet adhered to the Institute of Medicine Dietary Reference Intake guidelines for healthy nutrient intake (https://ods.od.nih.gov/HealthInformation/nutrientrecommendations.aspx) and was formulated by Brigham and Women’s Hospital Center for Clinical Investigation nutrition research unit. All food and beverages were provided for the duration of the study. Intake of alcoholic beverages was not permitted. Calories were adjusted to compensate for any complaints of hunger or satiety or changes in body weight.

During the 3-day tracer study, participants consumed a prescribed low-leucine diet (38% fat, 56% carbohydrate, 6% protein, ~2 g of leucine per 2,000 kcal) in order to promote the incorporation of tracer leucine, as opposed to dietary leucine, into newly synthesized proteins. On the morning of the tracer infusion, participants consumed a low-leucine breakfast between 6 and 8 am before being admitted to Brigham and Women’s Hospital Center for Clinical Investigation at 9 am. At 10 am, participants received an intravenous bolus injection of the stable isotope tracer D3-Leu (Cambridge Isotope Laboratories, Inc. catalog DLM-1259-MG) at a concentration of 10 mg/kg over 10 minutes. Blood was sampled immediately before the injection (time 0 hour, 10 am) and at 0.5, 1, 1.5, 2, 3, 4, 6, 10, 12, 14, 16, 18, 22, 46, and 70 hours postinfusion. Participants consumed a low-leucine lunch, dinner, and snack following the 2-hour (12 pm), 8-hour (6 pm), and 10-hour (8 pm) blood draws, respectively, and breakfast the following morning at 7:15 am, before the 22-hour (8 am) blood draw. All meals were consumed within 30 minutes. After collection of the 22-hour sample, participants were discharged. The 46- and 70-hour blood samples were collected at the ambulatory Center for Clinical Investigation.

### Total plasma leucine enrichment quantification

Total plasma leucine (D3-Leu labeled and endogenous) was isolated from 0.2 mL of plasma from time points 0, 1, 2, 3, 4, 8, 10, 12, 14, 16, 18, 22, 46, and 70 hours postinfusion using an AG 50W-X8 cation exchange resin (Bio-Rad, catalog 1421431). The isolated amino acids were then dried under nitrogen, derivatized to heptafluorobutyric acid esters, and measured using gas chromatography–mass spectrometry (Agilent 6890 GC, 5973 MS). Total plasma leucine tracer enrichment was quantified for participants 1 to 6 by taking the area under the curve of the tracer (D3-Leu) divided by the area under the curve of total plasma leucine (D3-Leu tracer + D0-Leu tracee).

### HDL isolation

Immediately after each blood sample collection, plasma was isolated by refrigerated centrifugation (2,500 rpm for 20 minutes at 4°C), aliquoted, and stored at –80°C. HDL was isolated from 1 to 14 time points per participant: participant 1: 0.5, 2, 4, 6, 12, 18, 22, 70 hours; participant 2: 1.5, 2, 3, 4, 6, 10, 12, 14, 16, 22, 70 hours; participant 3: 0, 2, 4, 6, 12, 18, 22, 70 hours; participant 4: 0, 0.5, 1, 1.5, 2, 4, 6, 8, 10, 12, 14, 22, 70 hours; participant 5: 0.5, 2, 4, 6, 12, 18, 22, 70 hours; participant 6: 0.5, 1, 1.5, 2, 3, 4, 6, 10, 12, 14, 16, 18, 22, 70 hours; participants 7–12: 2 hours.

The specific time points for each participant were chosen before HDL isolation and data analysis and varied across participants due to sample availability (samples from this same clinical study were analyzed in previous publications; refs. 7, 51, 52, 60–62). For each time point, HDL was purified from 1 mL of plasma by a 16-hour overnight incubation with 1.4 mL of anti-APOA1 immunoglobulin sepharose 4B resin (10 mg antibody per 1 mL of resin, Academy Biomedical, catalog 11A-G1 Resin) in 20 mL Econo-Pac chromatography columns (Bio-Rad, catalog 7321010). The unbound, non–APOA1-containing fraction was collected by gravity flow, and the bound, APOA1-containing fraction was eluted by 3 M NaSCN (MilliporeSigma, catalog 251410).

### Native gel electrophoresis and proteolysis

Immediately after isolation, APOA1-HDL was separated by size using nondenaturing polyacrylamide gel electrophoresis on a 4% to 30% gradient gel (Jule Inc., catalog 430N15HLC10G) run at 15 mA for 16 hours. A molecular weight standard from the GE Healthcare calibration kit (catalog 17-0445-01) was run alongside the samples. After completion of the run, the gel was stained for 1 to 2 hours in Coomassie blue (Invitrogen, Thermo Fisher Scientific, catalog LC6065). Portions of the gel corresponding to each HDL size were excised: above 12.2 nm, the largest HDL size, alpha0; between 12.2 and 9.5 nm, alpha1; between 9.5 and 8.3 nm, alpha2; between 8.2 and 7.2 nm, alpha3; and the band at 7.1 nm, prebeta. Additionally, we identified small amounts of APOA4 and APOA1 in the portion of the gel below the 7.1 nm prebeta protein band. We included this sixth size fraction in our analysis, and refer to it throughout as prebeta-b, for below prebeta. The excised gel pieces were proteolyzed using trypsin for 4 hours at 37°C using a standard protocol (63), with the exception that the alkylation step was omitted to increase throughput, and the final wash step (before digestion) comprising 2:1 ammonium bicarbonate/acetonitrile was done overnight to ensure complete removal of Coomassie stain. Peptides were resuspended in 5% acetonitrile and 0.5% formic acid dissolved in mass spectrometry–grade water.

### APOA4 and APOA1 protein concentrations in plasma and total HDL

Levels of APOA4 and APOA1 in plasma and on HDL were measured in participants 1 to 12 using the 2-hour time point by ELISA. The anti-human APOA4 antibodies used for coating (rabbit polyclonal, Proteintech, catalog 17996-1-AP) and detection (rabbit polyclonal, MilliporeSigma; catalog SPR687CA) were run at a working concentration of 2 and 1 μg/mL, respectively. The anti-human APOA1 antibodies used for coating (goat polyclonal, catalog 11A-G2b) and detection (goat polyclonal anti-APOA1 biotin conjugate, catalog 11B-G2b) were from Academy Biomedical and used at a working concentration of 5 and 1 μg/mL, respectively.

### Absolute quantification of protein pool sizes across 6 HDL sizes

In vitro synthesized peptide standards were used to quantify the pool size (mg of protein in a given plasma pool) of APOA1 and APOA4 across the 6 HDL sizes in participants 1 to 6. Two peptide standards were used to quantify APOA1 (THLAPYSDEL[R-labeled], ATEHLSTLSE[K-labeled]) and 1 for APOA4 (TQVNTQAEQL[R-label]) (New England Peptides). Proteins were quantified by the absolute amino acid method (New England Peptides). Arginines were labeled with 13C6,15N4 and lysines with 13C6,15N2. The peptide standards were chosen based on the following criteria: 1) fully cleaved, 2) devoid of methionines and cysteines, 3) highest ionization/signal intensity relative to other observed peptides passing criteria 1 and 2, and 4) not reported to be posttranslationally modified (UniProt).

We estimated the appropriate spike-in amount for the peptides by determining the linear range of ionization for both standard and sample-derived peptides (7). Due to the large dynamic range of APOA1 and APOA4 across the HDL sizes, we used 2 spiking mixtures. The first mixture contained a final on-column amount of 100 fmol of each APOA1 peptide and 1 fmol for the APOA4 peptide standard. The second mixture contained the same 3 peptides at a 10-fold lower on-column concentration. Peptide abundance was quantified for the 2- and 4-hour time points (4 quantification replicates), and Skyline (https://skyline.gs.washington.edu) (64) was used for quantification of the area under the curve of each peptide.

We calculated pool size for APOA1 and APOA4 in the 6 HDL sizes. The fmol on-column values (average of 4 replicates) for each protein per size fraction were summed to determine the total fmol of protein on HDL. The fmol value for each size was then divided by the HDL total (sum of fmol values across the 6 HDL sizes) to determine the fraction of total HDL protein in each HDL size. The total APOA1 mg/dL concentration on HDL and total APOA4 mg/dL concentrations on HDL, as determined by ELISA (see Methods, “APOA4 and APOA1 protein concentrations in plasma and total HDL,” above) were then multiplied by the fraction of protein in each HDL size to determine the concentration of protein in each HDL size. The mg/dL protein concentration per size was then multiplied by the total plasma volume to determine the protein pool size per HDL size. Plasma volume per participant was calculated: plasma volume in dL = (ideal body weight in kg × 0.44) + (excess body weight in kg × 0.1) (65).

### Mass spectrometry

For this study, we used 2 mass spectrometers. The Q Exactive Quadrupole Orbitrap (Thermo Fisher Scientific) was used for absolute quantification of APOA1 and APOA4 pool sizes as well as the relative quantification of the HDL proteome across the 6 HDL sizes. We utilized the Orbitrap Fusion Lumos (Thermo Fisher Scientific) for tracer enrichment quantification of APOA4 and APOA1 across 6 HDL sizes. Both instruments were coupled to an EASY-nLC 1000 HPLC pump (Thermo Fisher Scientific). The Q Exactive was fronted with a Nanospray FLEX ion source and the Lumos with an EASY-Spray ion source (Thermo Fisher Scientific).

#### Q exactive.

Peptides were separated using a dual-column setup: an Acclaim PepMap RSLC C18 trap column, 75 μm × 20 mm, and an Acclaim PepMap RSLC C18 analytical column, 75 μm × 250 mm (Thermo Fisher Scientific). The analytical gradient was run at 250 nL/min from 5% to 18% solvent B (acetonitrile/0.1% formic acid) for 10 or 30 minutes, followed by 5 minutes of 95% solvent B. Solvent A was 0.1% formic acid.

For absolute quantification of APOA1 and APOA4 across 6 HDL sizes in participants 1 to 6, the Q Exactive was set to selected ion monitoring for the scan range of *m/z* 400–1,000. For data-dependent acquisition of the HDL proteome, the Q Exactive was set to 140,000 resolution, and the top 10 precursor ions (with a scan range of *m/z* 380–1,500) were subjected to higher-energy collisional dissociation (HCD), with a collision energy 25% ± 10%, isolation width 3 *m/z*, and 17,500 resolution for MS/MS scans. The proteome was analyzed in the 2-hour time point for participants 1 to 12. The proteome of each HDL size was determined and combined into 1 data set per participant. We only included stable, reliably detected proteins that were identified by 3 or more unique peptides per participant data set and that were detected in all 12 participants.

#### Lumos.

Peptides were separated using a dual-column setup: an Acclaim PepMap RSLC C18 trap column, 75 μm × 20 mm, and a heated EASY-Spray column (45°C), 75 μm × 250 mm (Thermo Fisher Scientific). The gradient flow rate was 300 nL/min from 8% to 25% solvent B (acetonitrile/0.1% formic acid) for 10 minutes, 25% to 95% solvent B for 2 minutes, followed by an additional 5 minutes of 95% solvent B. Solvent A was 0.1% formic acid. Data-dependent acquisitions on the Lumos provided retention times of APOA4 and APOA1 peptides used for targeted mass spectrometry (Supplemental Table 2). The instrument was set to 120,000 resolution, and the top N precursor ions in a 3-second cycle time (within a scan range of *m/z* 375–1,500) were subjected to HCD (collision energy 30%) for peptide sequencing using a 30,000 resolution setting. The parallelization feature was enabled (automatic gain control [AGC] target, 1.0 × 10^5^; maximum injection time, 54 ms).

Targeted mass spectrometry was used to determine APOA1 and APOA4 enrichment across the 6 HDL sizes in participants 1 to 6. Targeted mass spectrometry was performed using the “targeted MS2 scan” module in scheduled mode. Dissociation was set to 30% HCD collision energy, and the targeted scans (*m/z* 150–1,000) were set to 240,000 resolution (AGC target 2.0 × 10^5^; maximum injection time, 502 ms). Each peptide’s targeted isolation window was centered on the average *m/z* of the M0 and 2HM3 peaks using a 4 Dalton isolation window, as previously described (7, 51). The enrichment of 2 to 6 peptide fragment ions was determined for 3 peptides for APOA1 and for 3 peptides for APOA4 (Supplemental Table 2). These peptides were selected based on the following criteria: 1) containing at least 1 leucine within the first 5 amino acids from either terminus and 2) consistent detection of D3-Leu tracer signal and enrichment across the size fractions and participants (7, 51, 60). The median enrichment across the fragment ions was then determined per peptide for each time point (0 to 70 hours postinfusion). The median enrichment values for the 3 APOA1 peptides and 3 APOA4 peptides for each time point were then averaged and used for compartmental modeling. The enrichment values for each peptide fragment ion and the median of the fragment ions per peptide across the 6 participants, 6 HDL size fractions, and study time course are summarized in Supplemental Table 3. The average APOA4 and APOA1 enrichment values used for kinetic modeling for all participants are shown in Supplemental Figures 1 and 2, respectively.

### Compartmental modeling

Compartmental modeling for APOA4 and APOA1 across 6 HDL sizes was performed using SAAM II software (Nanomath LLC). The previously published APOA4 model that contained only the alpha3 and prebeta sizes (7) was expanded upon to include APOA4 on alpha0, 1, 2, and prebeta-b. The previously published APOA1 model that contained the alpha0, 1, 2, 3, and prebeta sizes (7, 8, 51, 52) was expanded upon to include prebeta-b.

Both models contain an input, a source, and 6 HDL size compartments. The input compartment is the plasma amino acid precursor pool (D3-Leu tracer enrichment in plasma) expressed as a forcing function that drives the appearance of D3-Leu tracer in the model. Each participant’s plasma D3-Leu tracer enrichment curve was used for both models. The source compartment accounts for the time necessary for D3-Leu–labeled protein to appear on each HDL size in plasma. A single compartment was used as the source compartment for the APOA1 model. A 3-compartment delay was used as the source compartment for APOA4. This 3-compartment source delay was necessary to account for the delayed but rapid ascending slope of APOA4 on the small HDL sizes. The pool size and tracer enrichment data were assigned to each HDL size compartment. Two additional compartments were included in the APOA1 model: 1) delay compartment connecting the source and prebeta and the source and prebeta-b. This may represent an extravascular delay processing compartment that included APOA1 prebeta and prebeta-b that has been secreted but is outside systemic circulation (7, 8, 52). 2) Compartment connecting alpha3 and prebeta and alpha3 and prebeta-b. This compartment may represent lipidated APOA1 that has been released from alpha3 and is used to generate prebeta and prebeta-b (7, 8, 52). In the APOA4 model, an additional compartment (the pre-large compartment) connecting the source to the larger sizes was necessary to account for the delayed appearance of APOA4 on the larger size fractions. For both models, a direct secretion pathway from the source into each size, or from the source through the pre-large compartment and then into each large HDL size in the case of APOA4, was required for satisfactory fitting. A removal pathway out of each HDL size was also included in both models. This pathway represents the removal of protein out of a given HDL size in circulation, such as by hepatic uptake or by protein transfer to a compartment not measured in our study (i.e., to APOB-containing lipoproteins or to APOA4-containing lipoproteins that do not have APOA1). In the APOA4 model, we also tested pathways between HDL sizes to determine whether APOA4 may transfer among the different HDL sizes, but no transfer pathways between sizes were detected.

The following kinetic parameters were calculated for APOA1 and APOA4 on each HDL size in 6 participants: 1) FCR, the fraction of a given plasma protein pool turned over per day was determined for each protein in each HDL size by taking the sum of the rate constants exiting that compartment. 2) PR, the amount (mg) of protein produced or transferred into each HDL size per day per kilogram of body weight. PR = FCR (pools/d) × pool size (mg)/body weight (kg). 3) System FCR, fraction of protein in the model system turned over per day (pools/d), was calculated by taking the system PR (mg/kg/d) divided by the system pool size (mg) × body weight (kg). 4) System PR, amount of protein (mg) entering the model system per day per kilogram of body weight, was determined by taking the sum of all fluxes (mg/kg/d) exiting the source compartment. 5) Flux of a protein from the source compartment into each HDL size, and from one HDL size to another (mg/kg/d). 6) Rate of removal (*k*) out of each HDL size by a given pathway (pools/d). Steady-state kinetics was assumed for APOA1 and APOA4. A detailed overview of HDL protein compartmental model development is outlined in the Supplemental Methods.

### Statistics

Results are presented as mean ± SD. Kinetics parameters (FCR, PR, and pool size) were compared between women and men by 2-tailed, unpaired *t* test, with *P* < 0.05 being statistically significant. Data graphs and tables were constructed in GraphPad Prism and Microsoft Excel and compiled in Microsoft PowerPoint. Heatmaps and hierarchical clustering were executed using Qlucore Omics Explorer software (https://qlucore.com).

### Study approval

All participants in this study gave written informed consent. This study was approved by the Institutional Review Board of Brigham and Women’s Hospital and Harvard T.H. Chan School of Public Health (IRB 2010P001743/BWH).

## Author contributions

ABA devised the hypothesis of separate origins for large and small APOA4-containing HDL, prepared HDL samples, performed compartmental modeling analysis, and wrote the manuscript. SAS, HH, and LHL acquired and analyzed mass spectrometric data; SAS performed manual quantification of enrichment data and edited the manuscript; HH performed absolute quantification analyses; and LHL analyzed the HDL proteome data. FMS and MA supervised the study, discussed the findings, and participated in writing the manuscript.

## Supplementary Material

Supplemental data

Supplemental table 3

Supplemental table 5

Supplemental table 6

## Version 1 04/24/2023

Electronic publication

## Version 2 05/27/2023

Ref. 60 now has elocation id
