# Supplementary material for: The distinct metabolism between large and small HDL indicates unique origins of human apolipoprotein A4
Source: JCI Insight. 2023 Apr 24;8(8):e162481. doi: 10.1172/jci.insight.162481 (PMC10243815; doi:10.1172/jci.insight.162481)
Supplement: Supplemental data [file jciinsight-8-162481-s237.pdf]

The distinct metabolism between large and small HDL indicates unique origins of human apolipoprotein A4

Allison B. Andraski, Sasha A. Singh, Hideyuki Higashi, Lang Ho Lee, Masanori Aikawa, and Frank M. Sacks

SUPPLEMENTAL MATERIAL

Supplemental Figures 1, 2

Supplemental Tables 1, 2, 4, 7

Supplemental Methods

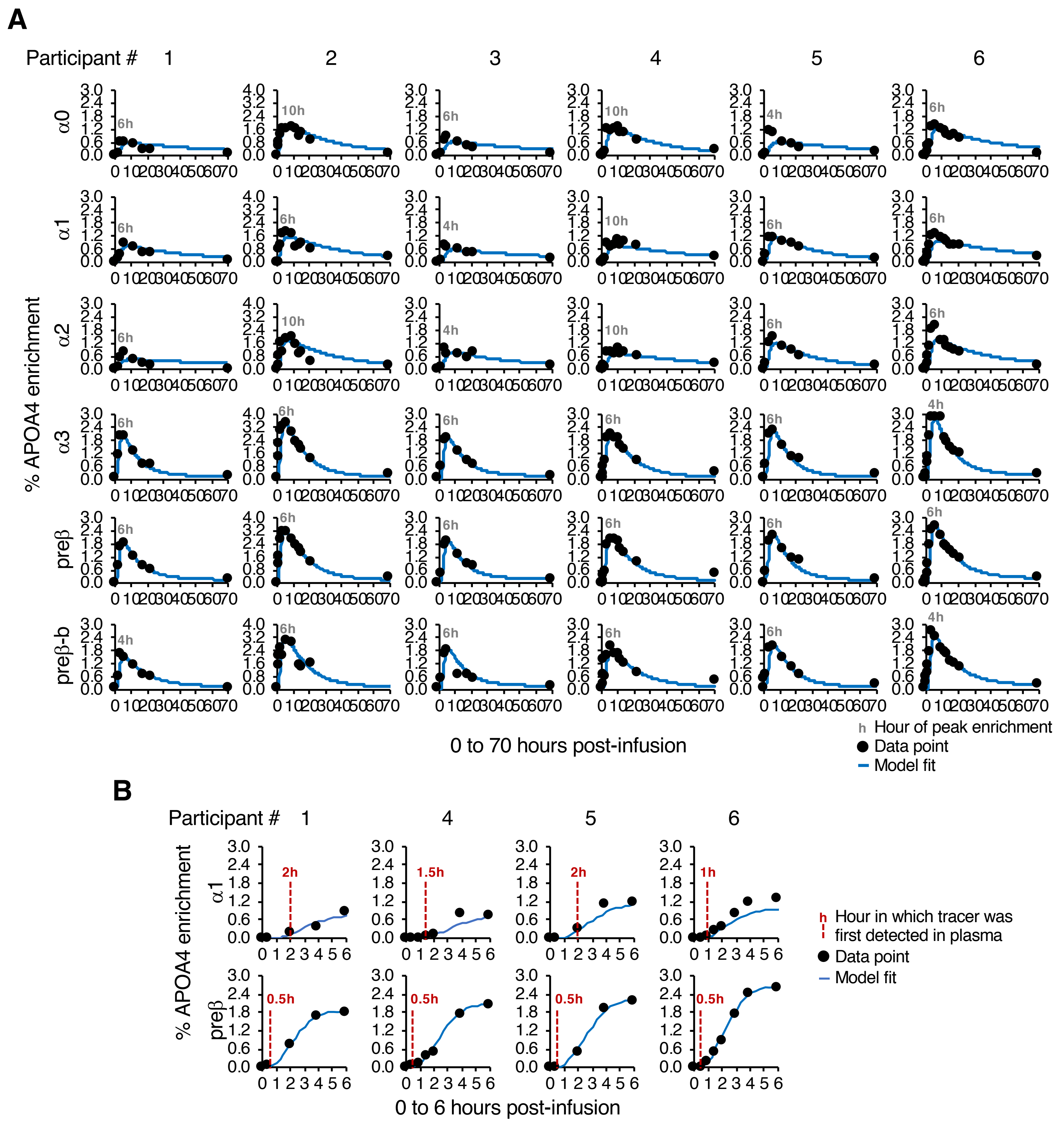

Supplemental Figure 1. APOA4 enrichment data and SAAM II-generated model fits for 6 participants. (**A**) Enrichment data and model fits for each participant across the 6 HDL sizes over the full time course (0-70 hrs). Time point indicated in grey above each enrichment curve is the hour of peak enrichment for each size fraction. (**B**) Zoom into the first 6 hours of the time course. Dashed red line and hour indicate the time in which APOA4 tracer was first detected on large and small HDL in plasma (alpha1 and prebeta are shown as representative large and small sizes, respectively). Participants 2 and 3 are not shown since the 0.5 hour time point was not monitored in these participants (the earliest time point monitored for participants 2 and 3 was 1.5 hours and 2 hours, respectively).

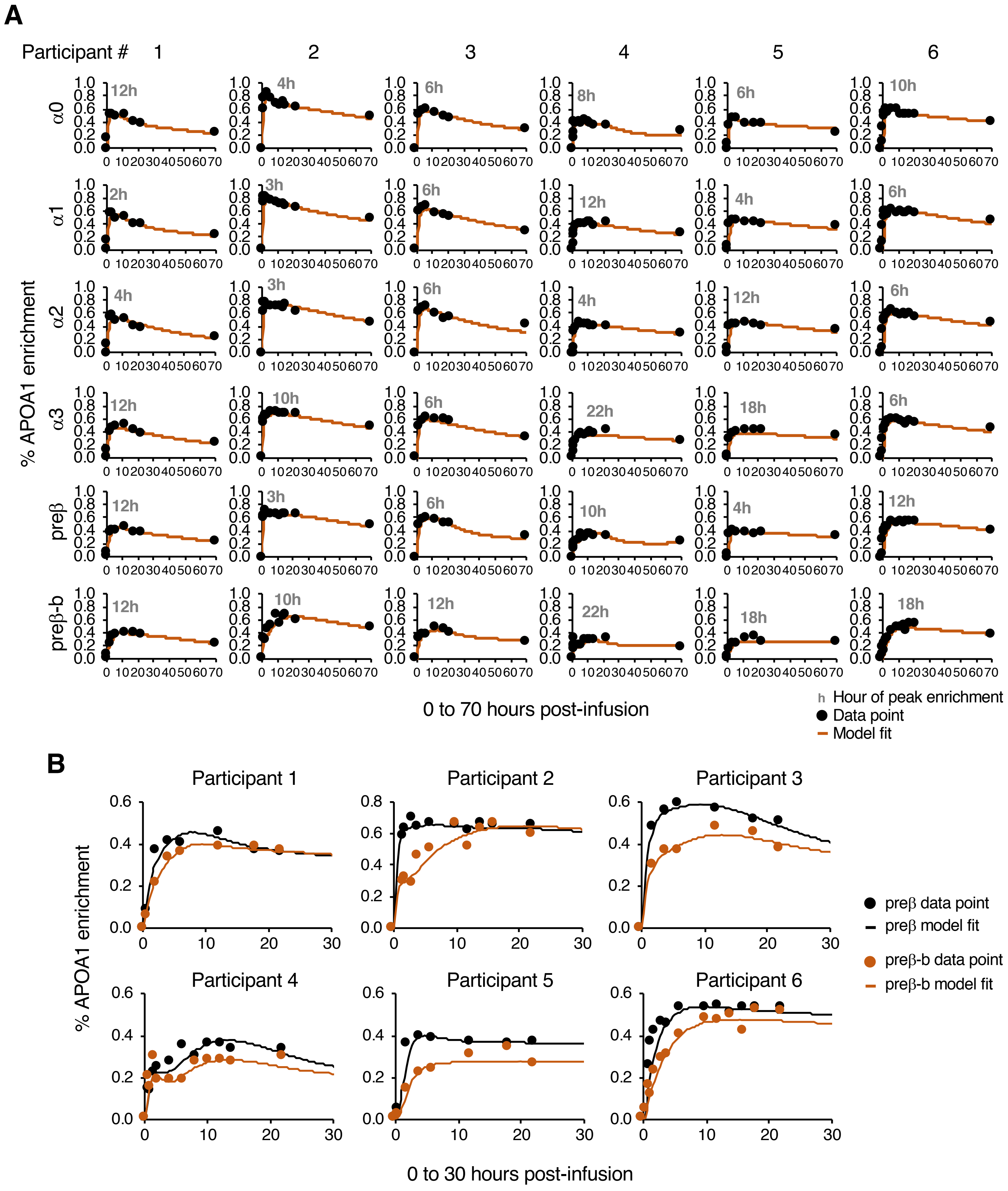

Supplemental Figure 2. APOA1 enrichment data and SAAM II-generated model fits for 6 participants. (**A**) Enrichment data and model fits for each participant across the 6 HDL sizes. Time points indicated in grey above each enrichment curve is the hour of peak enrichment for each size fraction. (**B**) Zoomed in overlay comparing prebeta and prebeta-b model fits in the 6 participants.

Participant ID#	Gender	Race	Age	Height (m)	IBW (kg)	Weight (kg)	BMI (kg/m^2)	TG (mg/dL)	Total-C (mg/dL)	LDL-C (mg/dL)	HDL-C (mg/dL)
1*	female	black	33	1.70	72.5	85.8	29.6	50	142	95	37
2*	male	white	39	1.85	85.9	110.3	32.1	138	176	103	45
3*	male	white	49	1.73	74.8	94.2	31.5	262	158	82	24
4*	male	black	48	1.75	76.9	91.7	29.8	64	171	116	42
5*	female	white	25	1.69	71.3	75.6	26.5	54	132	73	48
6*	female	white	40	1.68	70.4	86.1	30.6	139	219	147	44
7	female	black	25	1.58	62.1	87.2	35.1	62	145	85	48
8	female	white	23	1.58	62.1	68.8	27.7	87	123	59	47
9	female	white	70	1.65	68.3	85.5	31.3	72	193	125	54
10	female	white	62	1.60	64.1	90.5	35.3	76	175	105	55
11	female	white	33	1.61	65.2	80.8	31	88	193	122	53
12	male	white	59	1.83	83.8	92.8	27.7	59	144	96	36
Mean (SD)	8 female<br>4 male	9 white<br>3 black	42 (16)	1.69 (0.09)	71.4 (7.9)	87.4 (10.3)	30.7 (2.7)	95.9 (60.1)	164 (28.6)	101 (24.5)	44.4 (8.8)

Supplemental Table 1. Clinical characteristics. BMI, body mass index; TG, triglyceride; total-C, total cholesterol, LDL-C, LDL-cholesterol; HDL-C, HDL-cholesterol; *, participant samples included in the APOA1 and APOA4 metabolism analysis.

Protein	Peptide sequence	*z*	Ion type	*m/z*	Label	Isolation *m/z*	RT (min)
APOA1	THLAPYSDELR	3	precursor	434.5543	light M0	435.5606	8.5 to 10.5
	THLAPYSDELR	3	precursor	436.5669	heavy M3		
	THL	1	b3	352.1979	light M0		
	THL	1	b3	355.2168	heavy M3		
	ELR	1	y3	417.2456	light M0		
	ELR	1	y3	420.2644	heavy M3		
	THLA	1	b4	423.2350	light M0		
	THLA	1	b4	426.2539	heavy M3		
	THLAP	1	b5	520.2878	light M0		
	THLAP	1	b5	523.3066	heavy M3		
	DELR	1	y4	532.2726	light M0		
	DELR	1	y4	535.2914	heavy M3		
	SDELR	1	y6	782.3679	light M0		
	SDELR	1	y6	785.3867	heavy M3		
	AKPALEDLR	3	precursor	338.1977	light M0	339.2040	7.5 to 9.5
	AKPALEDLR	3	precursor	340.2103	heavy M3		
	LR	1	y2	288.2030	light M0		
	LR	1	y2	291.2218	heavy M3		
	EDLR	1	y4	532.2726	light M0		
	EDLR	1	y4	535.2914	heavy M3		
	VKDLATVYYVDVLK	3	precursor	488.2887	light M0	489.2950	13 to 15
	VKDLATVYYVDVLK	3	precursor	490.3013	heavy M3		
	DVLK	1	y4	474.2922	light M0		
	DVLK	1	y4	477.3111	heavy M3		
	VDVLK	1	y5	573.3606	light M0		
	VDVLK	1	y5	576.3795	heavy M3		
	YVDVLK	1	y6	736.4240	light M0		
	YVDVLK	1	y6	739.4428	heavy M3		
	VKDLAT	1	b6	628.3665	light M0		
	VKDLAT	1	b6	631.3853	heavy M3		
APOA4	LVPFATELHER	3	precursor	437.9067	light M0	438.9130	11 to 13
	LVPFATELHER	3	precursor	439.9193	heavy M3		
	LHER	1	y4	554.3045	light M0		
	LHER	1	y4	557.3234	heavy M3		
	ELHER	1	y5	683.3471	light M0		
	ELHER	1	y5	686.3659	heavy M3		
	TELHER	1	y6	784.3948	light M0		
	TELHER	1	y6	787.4136	heavy M3		
	ATELHER	2	y7	428.2196	light M0		
	ATELHER	2	y7	429.7290	heavy M3		
	FATELHER	2	y8	501.7538	light M0		
	FATELHER	2	y8	503.2632	heavy M3		
	ISASAEELR	2	precursor	488.2589	light M0	489.0136	8 to 10
	ISASAEELR	2	precursor	489.7683	heavy M3		
	ELR	1	y3	417.2456	light M0		
	ELR	1	y3	420.2644	heavy M3		
	EELR	1	y4	546.2882	light M0		
	EELR	1	y4	549.3070	heavy M3		
	SAEELR	1	y6	704.3573	light M0		
	SAEELR	1	y6	707.3762	heavy M3		
	ALVQQMEQLR	2	precursor	608.3293	light M0	609.8388	11.5 to 13.5
	ALVQQMEQLR	2	precursor	611.3482	heavy M3		
	QLR	1	y3	416.2616	light M0		
	QLR	1	y3	419.2804	heavy M3		
	EQLR	1	y4	545.3042	light M0		
	EQLR	1	y4	548.3230	heavy M3		
	MEQLR	1	y5	676.3447	light M0		
	MEQLR	1	y5	679.3635	heavy M3		
	QMEQLR	1	y6	804.4032	light M0		
	QMEQLR	1	y6	807.4221	heavy M3		

Supplemental Table 2. APOA1 and APOA4 peptides used to quantify tracer enrichment by targeted mass spectrometry. *z,* charge. *m/z*, mass-to-charge of the light M0 (D0-Leu) and heavy M3 (D3-Leu) fragment ions. Isolation *m/z*, the average mass of the light and heavy ions used as the center of the 4 Da targeted isolation window. RT, retention time range for each peptide.

		APOA4			APOA1		
HDL size	Participant #	FCR (pools/day)	PR (mg/kg/day)	PS (mg)	FCR (pools/day)	PR (mg/kg/day)	PS (mg)
α0	1 - female	0.60	0.003	0.42	4.48	2.08	41.24
	2 - male	1.02	0.066	7.09	3.22	1.07	36.61
	3 - male	0.68	0.006	0.74	0.99	0.32	29.62
	4 - male	1.05	0.049	4.05	3.94	1.56	34.70
	5 - female	0.54	0.007	0.94	10.13	1.83	13.24
	6 - female	0.87	0.018	1.69	4.07	1.28	26.29
	mean (SD)	0.79 (0.22)	0.025 (0.026)	2.49 (2.61)	4.47 (3.04)	1.36 (0.62)	30.28 (9.86)
	P-value	0.19	0.22	0.25	0.21	0.17	0.50
α1	1 - female	0.84	0.010	1.06	2.12	14.37	601.89
	2 - male	0.87	0.058	7.37	1.66	7.50	495.50
	3 - male	0.62	0.011	1.64	0.57	2.89	459.72
	4 - male	0.58	0.033	4.98	0.55	1.70	272.80
	5 - female	1.10	0.070	4.65	0.29	3.67	921.38
	6 - female	0.67	0.035	4.45	0.47	3.59	639.18
	mean (SD)	0.78 (0.20)	0.036 (0.024)	4.02 (2.33)	0.94 (0.76)	5.62 (4.71)	565.08 (216.87)
	P-value	0.32	0.85	0.57	0.96	0.49	0.07
α2	1 - female	0.46	0.004	0.82	0.65	13.03	1771.71
	2 - male	1.00	0.058	6.40	0.33	7.60	2514.78
	3 - male	0.90	0.026	2.68	1.02	19.24	1709.82
	4 - male	0.55	0.017	2.74	0.30	3.61	1058.91
	5 - female	1.19	0.056	3.44	0.44	16.74	2754.21
	6 - female	0.97	0.077	6.60	0.36	10.55	2435.12
	mean (SD)	0.84 (0.28)	0.040 (0.028)	3.78 (2.28)	0.52 (0.28)	11.80 (5.79)	2040.76 (637.99)
	P-value	0.83	0.67	0.89	0.82	0.57	0.34
α3	1 - female	2.92	0.25	7.70	0.56	8.40	1341.32
	2 - male	2.32	1.10	52.03	0.30	6.17	2249.77
	3 - male	2.83	0.59	18.94	0.54	7.81	1324.46
	4 - male	1.94	0.76	34.39	0.23	9.74	3688.55
	5 - female	2.69	0.92	25.12	0.24	6.84	2106.91
	6 - female	2.46	3.70	125.68	0.34	6.30	1559.61
	mean (SD)	2.52 (0.37)	1.22 (1.25)	43.98 (42.73)	0.37 (0.14)	7.54 (1.38)	2045.11 (894.09)
	P-value	0.34	0.52	0.68	0.88	0.59	0.39
preβ	1 - female	2.52	5.53	195.07	3.52	7.39	187.04
	2 - male	2.12	8.11	420.10	5.96	5.49	101.19
	3 - male	2.83	7.89	253.43	1.40	0.49	32.05
	4 - male	1.94	5.98	271.21	12.61	7.69	53.59
	5 - female	2.64	15.05	417.43	2.76	1.72	45.64
	6 - female	2.24	17.47	651.94	1.64	1.77	89.69
	mean (SD)	2.38 (0.34)	10.01 (5.01)	368.20 (166.24)	4.65 (4.23)	4.09 (3.16)	84.86 (56.63)
	P-value	0.61	0.28	0.51	0.34	0.76	0.41
preβ-b	1 - female	1.98	0.014	0.61	3.84	3.60	83.51
	2 - male	2.12	0.035	1.79	13.97	1.24	9.74
	3 - male	2.74	0.026	0.86	1.33	0.15	9.94
	4 - male	1.56	0.029	1.63	18.28	4.18	20.10
	5 - female	2.39	0.033	1.02	0.17	0.04	17.63
	6 - female	2.02	0.030	1.22	0.28	0.05	15.54
	mean (SD)	2.14 (0.40)	0.028 (0.008)	1.19 (0.45)	6.31 (7.84)	1.54 (1.88)	26.08 (28.44)
	P-value	0.98	0.56	0.24	0.19	0.73	0.37
system	1 - female	2.52	5.82	205.68	0.65	29.47	4026.70
	2 - male	2.09	9.42	494.78	0.33	16.17	5407.59
	3 - male	2.79	8.54	278.29	0.59	22.95	3565.61
	4 - male	1.89	6.86	319.00	0.28	16.32	5128.66
	5 - female	2.61	16.14	452.60	0.28	22.24	5859.01
	6 - female	2.25	21.34	791.58	0.36	20.37	4765.42
	mean (SD)	2.36 (0.34)	11.35 (6.08)	423.65 (210.17)	0.41 (0.16)	21.25 (4.95)	4792.17 (862.69)
	P-value	0.55	0.31	0.57	0.85	0.20	0.83

Supplemental Table 4. Kinetic parameters of APOA1 and APOA4 across 6 HDL sizes. FCR, fractional catabolic rate; PR, production rate; PS, pool size. *P*-value, kinetic parameters for each protein in each HDL size were compared between females and males by two-tailed, unpaired *t*-test (with unequal variance).

Gene name	UniProt entry #	Protein name	Average protein abundance (mean ± SD) for each protein detected in preβ-b (n=1 to 12 participants)
IGHM	P01871-2	Isoform 2 of Ig mu chain C region	-
FN1	P02751; P02751-15	Fibronectin	262,000 ± 145,434 (n=10)
A2M	P01023	Alpha-2-macroglobulin	892,500 ± 571,882 (n=8)
CFH	P08603	Complement factor H	-
APOB	P04114	Apolipoprotein B-100	210,000 (n=1)
C1QC	P02747	Complement C1q subcomponent subunit C	5,411,111 ± 1,678,871 (n=9)
LGALS3BP	Q08380	Galectin-3-binding protein	-
CLU	P10909-2	Clusterin	5,355,454 ± 2,052,760 (n=11)
FGB	P02675	Fibrinogen beta chain	1,082,727 ± 497,555 (n=11)
FGG	C9JC84	Fibrinogen gamma chain	17,883,333 ± 14,348,571 (n=12)
IGLC2	P0CG05	Ig lambda-2 chain C regions	520,000 (n=1)
FGA	P02671	Fibrinogen alpha chain	23,833,333 ± 14,529,489 (n=12)
HP	P00738	Haptoglobin	743,333 ± 718,850 (n=6)
HBB	P68871	Hemoglobin subunit beta	300,000 (n=1)
C1R	P00736	Complement C1r subcomponent	556,666 ± 332,014 (n=3)
APOL1	O14791-2	Apolipoprotein L1	722,727 ± 773,266 (n=11)
PLTP	B3KUE5	Phospholipid transfer protein	-
APOM	O95445	Apolipoprotein M	-
APOE	P02649	Apolipoprotein E	14,649,166 ± 11,707,895 (n=12)
ITIH2	P19823	Inter-alpha-trypsin inhibitor heavy chain H2	790,000 ± 181,934 (n=3)
C3	P01024	Complement C3	2,640,000 ± 1,521,578 (n=12)
GPLD1	P80108	Phosphatidylinositol-glycan-specific phospholipase D	-
CETP	P11597	Cholesteryl ester transfer protein	-
SAA4	P35542	Serum amyloid A-4 protein	782,500 ± 156,677 (n=12)
APOA2	V9GYM3	Apolipoprotein A-II	6,836,363 ± 4,517,139 (n=11)
APCS	P02743	Serum amyloid P-component	-
CFI	E7ETH0	Complement factor I light chain	-
APOA1	P02647	Apolipoprotein A-I	424,283,333 ± 385,245,446 (n=12)
APOC4-APOC2	K7ER74	Protein APOC4-APOC2	-
AGT	P01019	Angiotensinogen	-
VTN	P04004	Vitronectin	3,875,454 ± 2,872,181 (n=11)
APOD	C9JF17	Apolipoprotein D	5,566,666 ± 3,148,255 (n=12)
APOC3	B0YIW2	Apolipoprotein C-III	7,133,333 ± 8,831,336 (n=9)
PON1	F5H4W9; P27169	Serum paraoxonase/arylesterase 1	257,333,333 ± 192,957,995 (n=12)
C4B	P0C0L5	Complement C4-B	2,700,000 ± 863,133 (n=5)
CNDP1	Q96KN2	Beta-Ala-His dipeptidase	-
TF	P02787	Serotransferrin	-
SERPINA3	P01011	Alpha-1-antichymotrypsin	755,000 ± 629,325 (n=2)
PROS1	P07225	Vitamin K-dependent protein S	320,000 ± 69,282 (n=4)
ITIH4	Q14624	Inter-alpha-trypsin inhibitor heavy chain H4	-
PLXDC2	Q6UX71	Plexin domain-containing protein 2	460,000 (n=1)
KNG1	P01042-2; P01042	Kininogen-1	792,000 ± 411,424 (n=5)
HPX	P02790	Hemopexin	-
SERPINC1	P01008	Antithrombin-III	580,000 (n=1)
C1S	P09871	Complement C1s subcomponent	-
LCAT	P04180	Phosphatidylcholine-sterol acyltransferase	-
CDH5	P33151	Cadherin-5	1,000,000 (n=1)
A1BG	P04217	Alpha-1B-glycoprotein	-
F5	P12259	Coagulation factor V	560,000 ± 254,990 (n=11)
AHSG	C9JV77	Alpha-2-HS-glycoprotein	-
SERPINA1	P01009	Alpha-1-antitrypsin	4,768,181 ± 6,644,517 (n=11)
APOA4	P06727	Apolipoprotein A-IV	13,566,666 ± 8,804,062 (n=12)
ALB	P02768	Serum albumin	8,625,000 ± 3,031,388 (n=12)

Supplemental Table 7. HDL proteins detected in prebeta-b. List of 53 proteins identified on HDL in 12 participants. The horizontal black lines separate proteins in the 5 protein clusters. The order in which the proteins are listed corresponds with **Figure 6A**. The right most column highlights the average abundance of prebeta-b proteins that were detected in 1 to 12 participants. The prebeta-b proteins detected in 9 to 12 participants in this list correspond with the proteins shown in **Figure 6B,C**. (n=), indicates the number of participates in which each protein was identified; -, HDL proteins not detected in prebeta-b.

SUPPLEMENTAL METHODS

Overview of compartmental modeling

The following section provides an overview of the compartmental modeling process used to determine the metabolism and kinetic parameters of HDL proteins across one or more HDL sizes in humans using SAAM II modeling software (The Epsilon Group, http://tegvirginia.com).

Model components – compartments and arrows: The basic structure of a compartmental model used to describe the metabolism of a given protein on a given HDL size typically contains 3 or more compartments - an input, source, and one or more HDL size compartments. An example of this basic model structure with 2 HDL sizes is shown below in **Supplemental Methods Figure 1**. Each compartment in the model represents a different physiological system or process. The input compartment (compartment 1) represents the plasma amino acid precursor pool. The total plasma D3-Leucine tracer enrichment data for a given participant is uploaded to this compartment and expressed as a forcing function that drives the appearance of D3-Leucine tracer in the model. The source compartment (compartment 2) accounts for the time it takes for labeled protein to appear on each HDL size in plasma. It represents the site of synthesis and secretion of the protein, and potentially any intermediate transfer steps (such as transfer to and from an APOB-containing lipoprotein) before it enters a given HDL size in plasma. The source compartment can be represented in the model as either a single compartment or a multi-compartment delay, and can be optimized to fit the time of appearance and ascending slope of the tracer-labeled protein as it appears on the HDL sizes in circulation. Compartments 3 and 4 represent alpha2 and alpha3 HDL, respectively, in plasma. Protein tracer enrichment data across the full metabolic study time course (i.e., 0-70 hours post-infusion in the present study) and pool size data are incorporated into each HDL size compartment (**Supplemental Methods Figure 1**). The pool size of a given protein in alpha2 and alpha3 are shown in the model as mg_3 and mg_4, respectively (**Supplemental Methods Figure 1**).

Pathways of tracer movement between and out of compartments are represented by arrows. Arrows from the source compartment into each HDL size represent the production of a given protein onto that HDL size. Arrows between compartments represent transfer of protein from one HDL size to another, and downward arrows represent protein removal out of that HDL size in circulation, such as by hepatic uptake or by protein transfer to a compartment not measured in the study (i.e., APOB-containing lipoproteins).

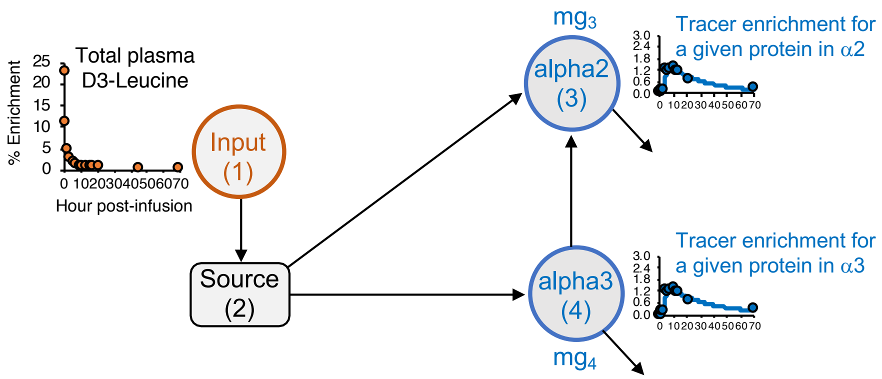

Supplemental Methods Figure 1. Example structure of a compartmental model used to determine the metabolism of a given HDL protein on specific HDL sizes in plasma. Circles and squares represent compartments, and numbers inside the compartment represent the compartment number. Total plasma D3-Leucine enrichment is uploaded to the input compartment (compartment 1, orange). Tracer enrichment and pool size data for a given HDL protein in a given HDL size are uploaded to the corresponding HDL size compartment (alpha2, compartments 3; alpha3, compartment 4; blue). Mg_x, the pool size in milligrams of a given protein in compartment x (mg_3, alpha2 pool size; mg_4, alpha3 pool size).

Model-generated rate constants and flux values: SAAM II uses the tracer and pool size data to generate a system of differential equations to estimate the rate and amount of protein entering and leaving each compartment per day for each pathway. A rate constant and flux value are estimated for each pathway (**Supplemental Methods Figure 2**). Rate constants ($K_{x,y}$) represent the rate at which a given protein is transferring into compartment x from compartment y in pools/day. For example, $K_{3,4}$ represents the rate at which a given protein is transferring to compartment 3 from compartment 4. $K_{0,y}$ represents the rate at which a given protein is being removed out of the model system (0) from compartment y. Flux values ($Flux_{x,y}$) indicate the amount of protein in milligrams (mg) transferring into compartment x from compartment y per day, and are calculated by taking the rate constant for that pathway ($K_{x,y}$) times the pool size of the protein in compartment y: $Flux_{x,y} = K_{x,y}*mg_y$. For instance, the flux of protein into

compartment 3 from compartment 4 and the flux of protein out of compartment 3 are equal to $Flux_{3,4} = K_{3,4}*mg_4$ and $Flux_{0,3} = K_{0,3}*mg_3$, respectively.

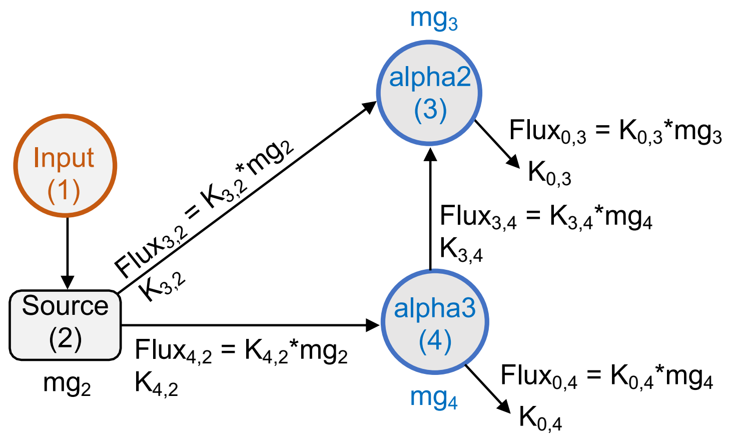

Supplemental Methods Figure 2. Model-generated rate constants and flux values for each pathway. $K_{x,y}$, rate of protein transfer into compartment x from compartment y by a given pathway (pools/day). Mg_x, the pool size in milligrams of protein in compartment x. $Flux_{x,y}$, amount of protein (mg) entering compartment x from compartment y by a given pathway per day (mg/day).

For these studies, we assume that the given HDL protein being modeled is in steady-state: the amount of protein entering a given HDL size per day is equal to the amount of that protein leaving that size, and the protein pool size in each compartment remains constant. Based on this steady-state premise, the flux of protein entering a given compartment must equal the flux of protein leaving the compartment. For example, in **Supplemental Methods Figure 2**, the flux of protein into alpha3 from the source ($Flux_{4,2}$) is equal to the flux of protein leaving alpha3 ($Flux_{0,4} + Flux_{3,4}$), and the flux of protein into alpha2 ($Flux_{3,2} + Flux_{3,4}$) is equal to the flux of protein leaving alpha2 ($Flux_{0,3}$).

Calculating kinetic parameters: The model-generated rate constants and fluxes are then used to calculate the fractional catabolic rate (FCR) and production rate for a given HDL protein on each HDL size. The FCR is the fraction of a given plasma protein pool turned over per day and is determined for each protein in each HDL size by taking the sum of the rate constants exiting that compartment. In **Supplemental Methods Figure 3**, the protein FCR out of alpha2 is equal to $K_{0,3}$ and the protein FCR out of alpha3 is equal to $K_{0,4} + K_{3,4}$ (light grey boxes). The production rate is the amount (mg) of protein produced or transferred into each HDL size per day per kg of body weight and is determined for a given protein on each HDL size by taking the sum of the fluxes into a given HDL size compartment divided by body weight. In **Supplemental Methods Figure 3**, the protein production rate into alpha3 is equal to

($Flux_{4,2}$ / kg of body weight), and the production rate into alpha2 is equal to ([$Flux_{3,2}$ + $Flux_{3,4}$] / kg of body weight) (dark grey boxes). Given that the system is in steady-state, and the flux of protein coming into each HDL size is equal to the flux of protein leaving that size, the production rate can also be calculated using the protein FCR and pool size for a given HDL size: Production rate = FCR (pools/day) x pool size (mg) / body weight (kg).

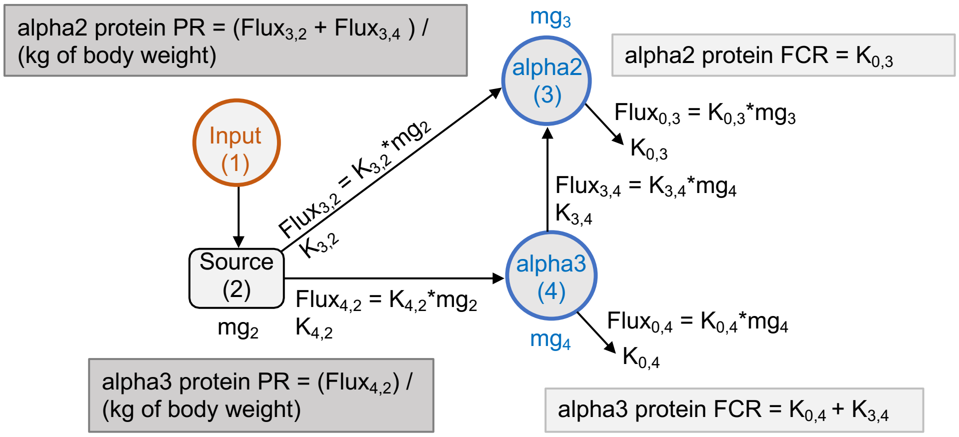

Supplemental Methods Figure 3. Using model-generated rate constants and flux values to determine the FCR and production rate of a given HDL protein on each HDL size. The FCR for a given protein out of each HDL size is determined by taking the sum of all rate constants exiting that size (pools/day, light grey boxes). The production rate (PR) for a given protein into each HDL size is determined by taking the sum of the flux values into each HDL size divided by body weight (mg/kg/day, dark grey boxes).

In addition to calculating the protein FCR and production rate out of and into each HDL size, respectively, we also determine the system FCR and the system production rate. The system FCR is the fraction of protein in the entire model system turned over per day (pools/day), and is calculated by taking the system production rate (mg/kg/day) divided by the system pool size (total mg of protein in all HDL sizes) x body weight (kg). The system production rate (mg/kg/day) is the total amount of protein (mg) entering the model system per day per kg of body weight, and is determined by taking the sum of all fluxes (mg/day) exiting the source compartment divided by body weight (kg).

APOA4 model and kinetic parameters: The basic compartmental model structure and principles outlined above were used to generate the APOA4 compartmental model and calculate the APOA4 kinetic parameters. **Supplemental Methods Figure 4** shows the APOA4 compartmental model

and highlights the rate constants and flux values used to calculate the FCR and production rate, respectively, of APOA4 on each HDL size.

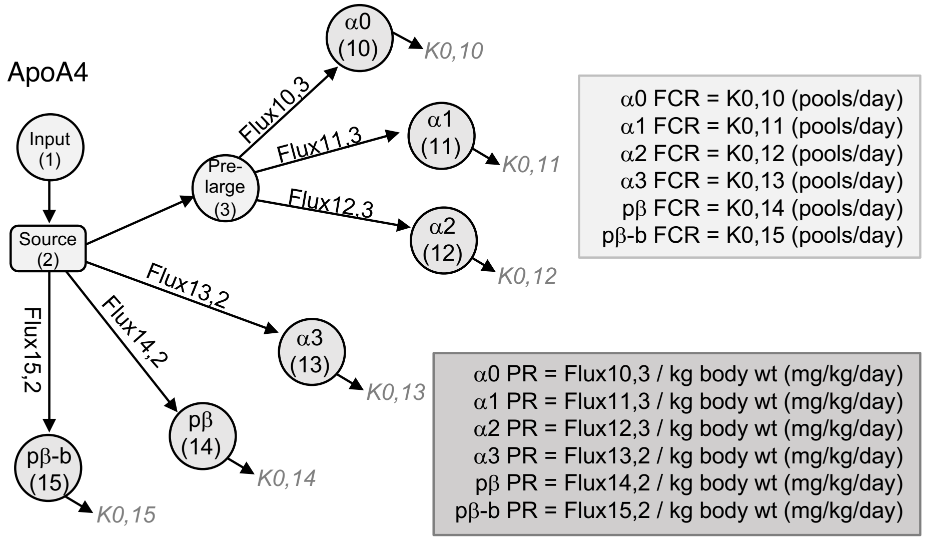

Supplemental Methods Figure 4. APOA4 compartmental model and the rate constants and flux values used to calculate FCR and production rate (PR), respectively, for APOA4 on each HDL size fraction in plasma.

APOA1 model and kinetic parameters: The basic compartmental model structure and principles outlined above were used to generate the APOA1 compartmental model and calculate the APOA1 kinetic parameters. A detailed description of APOA1 model development has been described previously (Mendivil et al. Arterioscler Thromb Vasc Biol. 2016).

REFERENCES

Mendivil CO, et al. Novel pathways of apolipoprotein A-I metabolism in high-density lipoprotein of different sizes in humans. *Arterioscler Thromb Vasc Biol.* 2016:57(4):714-28.
